# Supplementary material for: Text embedding models yield detailed conceptual knowledge maps derived from short multiple-choice quizzes
Source: Nat Commun. 2026 Mar 24;17:2055. doi: 10.1038/s41467-026-69746-w (PMC13013618; doi:10.1038/s41467-026-69746-w)
Supplement: Supplementary file 1 — Supplementary Information [file 41467_2026_69746_MOESM1_ESM.pdf]

Supplementary Information

Supplementary tables

Supplementary Table 1: Question pool. Each participant completed three quizzes consisting of 13 multiple-choice questions randomly selected from the question pool. Each quiz included 5 questions about the *Four Fundamental Forces* lecture (FFF), 5 questions about the *Birth of Stars* lecture (BoS), and 3 questions about general physics knowledge (GPK). Each question appeared on at most one quiz (for each participant).

ID	Question set	Question	Correct response	Alternative 1	Alternative 2	Alternative 3
1	FFF	Why is the gravitational attraction between you and your computer too small for you to notice?	Neither you nor your computer has enough mass to cause a noticeable gravitational attraction	You and your computer are too close for the gravitational attraction to be significant	Humans are too small to detect the force of gravity	The gravitational attraction between you and your computer is disrupted by the larger gravitational field generated by the earth
2	FFF	Which of the following is an example of the Weak Interaction?	A neutron in a radioactive Cesium atom is converted into a proton, leading to the release of a few particles	Light from the sun collides with a satellite orbiting Earth and exerts a small push on the satellite	Two protons bound together in a Helium nucleus resist separation despite a repulsive electromagnetic force acting on them	A distant galaxy exerts a small but detectable gravitational pull on the Earth
3	FFF	Roughly how many times stronger is the Weak Interaction than gravity?	10,000,000,000,000,000,000,000,000	10	1,000,000	The Weak Interaction is less strong than gravity
4	FFF	Why don't you and your computer experience any attraction or repulsion due to the Weak Interaction?	The weak interaction only acts over extremely small distances	The weak interaction between you and your computer is counteracted by the other forces	You and your computer have no net charge	Neither you nor your computer has enough mass to induce a significant Weak Interaction
5	FFF	Which of the following is a difference between gravity and the electromagnetic force?	Gravity is only ever attractive while the electromagnetic force can both attract and repel	Gravity is a much more powerful force than electromagnetism	Gravity can only act over large distances while the electromagnetic force can act over large and small distances	The electromagnetic force can only act over small distances while gravity can act over small or large distances
6	FFF	Electricity and magnetism can be shown to be two cases of the same force if we:	View them in different frames of reference	Switch which charges we call positive and which charges we call negative	Consider both the effects over small distances and the effects over large distances	Consider both the attractive and repulsive properties of the two forces
7	FFF	Which of the following are the primary two fundamental forces acting in opposition between the positively-charged protons in an atom's nucleus?	The Strong Force and the Electromagnetic Force	Gravity and the Weak Interaction	Gravity and the Electromagnetic Force	The Strong Force and the Weak Interaction
8	FFF	Why does the universe have a very uneven distribution of mass but a relatively equal distribution of charge?	Positive and negative charges cancel out and become a neutral charge when they combine while masses only grow larger as they combine	Masses tend to repel while charges tend to attract	Masses tend to attract while charges tend to repel	The gravitational interaction acting between masses is stronger than the electromagnetic interaction acting between charges
9	FFF	In your body, there are a tremendous amount of negatively-charged electrons. Your computer also contains a huge number of negatively-charged electrons. We know that like charges repel, but you and your computer are not repelled apart. Which of the following explains why?	The electrons' negative charges are balanced by the positive charges of an equal number of protons	An attractive gravitational force balances out this repulsion	The Electromagnetic force only acts over very small distances	The Electromagnetic force only acts over very large distances
10	FFF	Which of the following is a similarity between the Weak Interaction and the Strong Force?	Both act only over very small distances	Both are stronger than the Electromagnetic force	Both are weaker than Gravity	Both are responsible for attractions between distant galaxies
11	FFF	Which force is stronger than the Electromagnetic Force?	Strong Force	Gravity	Weak Interaction	Electromagnetic Force is the strongest
12	FFF	Roughly how many times stronger is the Strong Force than gravity?	10ˆ38	100	10ˆ18	The Strong Force is weaker than gravity
13	FFF	Which of the following would have to be true for the Weak Interaction to cause repulsion or attraction between two objects?	The objects would have to be extremely close to each other	The objects would have to have the same mass	The objects would have to be extremely far away from each other	The objects would have to have different masses
14	FFF	Which force keeps us from jumping off of Earth?	Gravity	Strong Force	Weak Interaction	Electromagnetic Force

Supplementary Table 1: (continued)

ID	Question set	Question	Correct response	Alternative 1	Alternative 2	Alternative 3
15	FFF	What does the Coulomb Force refer to?	The repulsion of objects with similar charge and the attraction of objects with different charge	The repulsion of objects with similar mass and the attraction of objects with different mass	The repulsion of objects with similar temperature and the attraction of objects with different temperature	The repulsion of objects with similar density and the attraction of objects with different density
16	BoS	Which of the following describes the effect of gravity on a cloud of atoms?	The atoms move to the center of the mass of the atoms	The atoms move away from the center of the mass of the atoms	The atoms spin around the center of the mass of the atoms	Gravity has no effect on a cloud of atoms
17	BoS	Which of the following occurs as a cloud of atoms gets more dense?	Temperature increases	Temperature decreases	Mass increases	Mass decreases
18	BoS	Which temperature does a cloud of hydrogen atoms approach as it gets denser in the process of becoming a star?	10 Million Kelvin	0 Kelvin	10,000 Kelvin	10 Billion Kelvin
19	BoS	Which of the following can overcome the Coulomb Force?	High temperature and high pressure	Low temperature and high pressure	High temperature and low pressure	Low temperature and low pressure
20	BoS	Which of the following prevents a star from collapsing as a result of gravity?	Energy released from the fusion of hydrogen atoms provides outward pressure	The fusion of hydrogen atoms decreases the temperature of the star	The gravitational pull of other stars nearby	The Weak Interaction
21	BoS	How are supermassive stars different from other stars?	Fusion occurs very fast	Fusion occurs very slow	Fusion occurs in the reverse order	Fusion does not occur at all
22	BoS	Which of the following is the FIRST product of two hydrogen atoms fusing together?	Deuterium	Oxygen	Helium	Beryllium
23	BoS	Once hydrogen atoms get close enough together, which of the following keeps them together?	The Strong Force	The Electromagnetic Force	Gravity	The Weak Interaction
24	BoS	When two nuclei fuse together, how does the mass of the combined nucleus compare to the mass of each of the original nucleus?	The mass of the combined nucleus is smaller	The mass of the combined nucleus is larger	The mass of the combined nucleus is the same	It is not possible for two nuclei to fuse together
25	BoS	If we say that our Sun is a main sequence star, what does that tell us about the Sun?	Hydrogen atoms in the Sun are fusing together and becoming Helium	The Sun is a supermassive star	The Sun does not experience the force of Gravity but does experience the Coulomb Force	The Sun is comprised of 10 million Hydrogen atoms
26	BoS	Which force would cause a massive cloud of hydrogen atoms to move together?	Gravity	Strong Force	Weak Interaction	Electromagnetic Force
27	BoS	Which of the following occurs as density increases?	Temperature increases	Volume increases	Mass increases	None of the above
28	BoS	Which of the following is a product of Hydrogen fusion?	Helium	Oxygen	Cesium	Carbon
29	BoS	Which of the following terms accurately describes the Sun?	Main sequence star	Supermassive star	Alternative sequence star	None of the above
30	BoS	Which of the following terms best describes a fusion reaction?	Ignition	Combustion	Decomposition	Displacement
31	GPK	Which of the following lists of particles is ordered from smallest to largest?	Electron, proton, nucleus, atom	Atom, electron, proton, nucleus	Electron, nucleus, atom, neutron	Neutron, nucleus, electron, atom
32	GPK	Which of the following defines what element an atom is?	Its number of protons	Its number of neutrons	Its number of electrons	Its mass
33	GPK	Suppose that in some atom, a proton is converted into a neutron. What changes as a result of this conversion?	The atom's element	The atom's mass (in atomic mass units)	The atom's velocity	The atom's density
34	GPK	Which of the following lists is ordered from smallest to largest?	Star, solar system, galaxy, universe	Galaxy, solar system, Milky Way, universe	Planet, galaxy, star, solar system	Earth, solar system, universe, galaxy
35	GPK	Which of the following are located in the nucleus of an atom?	Protons and neutrons	Only protons	Only electrons	Neutrons and electrons
36	GPK	Which of the following has the least mass?	An electron	A proton	A neutron	A hydrogen atom
37	GPK	What percent of an atom's space does its nucleus occupy?	Less than 1%	10%	50%	More than 90%
38	GPK	In the famous equation attributed to Albert Einstein, E = mcˆ2, what does the letter "m" represent?	Mass	Momentum	Moment of inertia	Moles

Supplementary Table 1: (continued)

ID	Question set	Question	Correct response	Alternative 1	Alternative 2	Alternative 3
39	GPK	If I were to heat up an inflated balloon, which of the the following would occur?	The balloon would expand	The balloon would shrink	None of these answers are correct	The balloon could expand or shrink depending on whether it's filled with air or helium gas

Supplementary Table 2: Topics. We fit a topic model with $k = 15$ topics to sliding windows parsed from the two course video transcripts (see *Constructing text embeddings of multiple lectures and questions*), and identified 14 topics with non-zero weights. The table displays the 10 top-weighted words (columns) from each of the topics (rows) discovered by the model. The weights of these words are shown in Supplementary Figure 1.

	Word rank									
**Topic**	**1**	**2**	**3**	**4**	**5**	**6**	**7**	**8**	**9**	**10**
1	star	helium	main	mass	atomic	sequence	get	energy	fuse	hydrogen
2	charge	force	mass	gravity	strong	attract	large	strength	distance	electromagnetic
3	huge	force	electromagnetic	macro	way	scale	concentration	apply	kind	charge
4	atom	dense	go	hydrogen	slow	get	huge	condense	mass	would
5	fusion	get	threshold	core	occur	mass	something	start	several	jupiter
6	enough	ignition	proton	force	get	close	nucleus	coulomb	fusion	would
7	energy	pressure	ignition	mass	little	keep	provide	fusion	get	hydrogen
8	proton	weak	neutron	interaction	one	go	nucleon	cesium	extra	get
10	huge	cloud	space	float	imagine	hydrogen	atom	say	distance	combine
11	one	hydrogen	helium	go	proton	neutron	keep	atomic	detail	fuse
12	gravity	force	weak	interaction	apply	strength	distance	ten	relative	next
13	force	go	electrostatic	call	charge	magnet	side	coulomb	know	different
14	force	atom	nucleus	electron	much	hydrogen	get	around	coulomb	charge
15	force	scale	gravity	start	weak	orbit	keep	fundamental	around	surprise

Supplementary Table 3: Lecture-specific questions and lecture excerpts. We used the text embeddings for each question about *Four Fundamental Forces* (FFF) and *Birth of Stars* (BoS) to identify corresponding matching intervals of the lectures. The text of the questions and the best-matching time intervals of the lecture transcripts are displayed (lecture times are noted in square brackets). Time courses of topic correlations for each question may be found in Supplementary Figures 3 and 4. Note that no matching lecture interval was found for Question 29.

ID	Question set	Question	Matched lecture transcript text
1	FFF	Why is the gravitational attraction between you and your computer too small for you to notice?	**[00:00.03–01:33.509]** what I want to do in this video is give a very high-level overview of the four fundamental forces four fundamental forces of the universe and I'm going to start with gravity I'm going to start with gravity and it might surprise some of you that gravity is actually the weakest of the four fundamental forces that's surprising because you say wow that's what keeps us glued not glued but it keeps us from jumping off the planet it's what keeps the moon in orbit around the earth the earth in orbit around the Sun the Sun in orbit around the the center of the Milky Way galaxy so it's it's a little bit surprising that it's actually the weakest of the forces and and that starts to make sense when you actually think about things on maybe more of a human scale or a molecular scale or even an atomic scale even on a human scale your computer monitor and you have some type of gravitational attraction but you don't notice it or your cell phone and your wallet there's gravitational attraction but you don't see them being drawn to each other the way you might see two magnets drawn to each other or repelled from each other and if you've got even a smaller scale you'll see that it matters even less we never even talk about gravity and chemistry although the gravity is there but at those scales the other forces really really really start to dominate so gravity's are weakest so if we move up a little bit from that we get and this is maybe the the hardest force for us to visualize or at least the hardest the least intuitive force for me is actually the weak force the weak sometimes called the weak interaction . . .
2	FFF	Which of the following is an example of the Weak Interaction?	**[01:22.439–03:05.1]** . . . this is maybe the the hardest force for us to visualize or at least the hardest the least intuitive force for me is actually the weak force the weak sometimes called the weak interaction and it's what's responsible for radioactive decay in particular beta minus and beta plus decay and just to give you an example of the actual weak interaction if I had some cesium 137 137 means it has 137 nucleons a nucleon is either a proton or a neutron you add up the protons and neutrons of cesium you get 137 and it is cesium because it has exactly 55 protons now the weak interaction is what's responsible for one of the neutrons essentially one of its corks flipping and turning into a proton and I'm not going to go into detail of what a cork is and all of that and the math can get pretty hairy but I just want to give you an example of what the weak interaction does so if one of these one of these neutrons turns into a proton then we're going to have one extra proton but we have the same number of nucleons instead of a extra Neutron here you now have an extra proton here and so now this is a different atom it is now barium and in that flipping it will actually emit it'll actually emit an electron and an anti-electron neutrino and I'm not going to go into the details of what an anti-electron neutrino is these are fundamental particles but this is just what that weak interaction is it's not something that's completely obvious to us it's not kind of this traditional things pulling or pushing away from each other like we would like we associate . . .
3	FFF	Roughly how many times stronger is the Weak Interaction than gravity?	**[02:52.38–04:43.73]** . . . anti-electron neutrino is these are fundamental particles but this is just what that weak interaction is it's not something that's completely obvious to us it's not kind of this traditional things pulling or pushing away from each other like we would like we associate with the other the other forces now the next strongest force and just to give a sense of how weak gravity is even relative to the weak interaction the weak interaction is 10 to the 25th times the strength of gravity times the strength of gravity and you might anything if this is so strong how come this doesn't operate on planets or us relative to the earth or the reason you know why doesn't this apply to intergalactic distances the way gravity does and the reason is the weak interaction really applies to very small distances very very small distances so it can be much stronger than gravity but only over very very and it really only applies on the sub atomic scale you go anything beyond that it kind of disappears as an actual force as an actual interaction now the next the next force up the hierarchy which is one that we are more familiar with it is something it's what actually dominates most of the chemistry that we deal with and electromagnetism that we deal with and that's the electro magnetic force we write it in magenta electro magnetic magnetic force and just to give a sense this is this is 10 to the 36 times the strength of gravity 10 to 36 times the strength of gravity so it kind of puts the weak force in its place it's 10 to the 12 times stronger than the weak force so these are huge numbers that we're talking about either this . . .
4	FFF	Why don't you and your computer experience any attraction or repulsion due to the Weak Interaction?	**[00:00.03–02:42.9]** what I want to do in this video is give a very high-level overview of the four fundamental forces four fundamental forces of the universe and I'm going to start with gravity I'm going to start with gravity and it might surprise some of you that gravity is actually the weakest of the four fundamental forces that's surprising because you say wow that's what keeps us glued not glued but it keeps us from jumping off the planet it's what keeps the moon in orbit around the earth the earth in orbit around the Sun the Sun in orbit around the the center of the Milky Way galaxy so it's it's a little bit surprising that it's actually the weakest of the forces and and that starts to make sense when you actually think about things on maybe more of a human scale or a molecular scale or even an atomic scale even on a human scale your computer monitor and you have some type of gravitational attraction but you don't notice it or your cell phone and your wallet there's gravitational attraction but you don't see them being drawn to each other the way you might see two magnets drawn to each other or repelled from each other and if you've got even a smaller scale you'll see that it matters even less we never even talk about gravity and chemistry although the gravity is there but at those scales the other forces really really really start to dominate so gravity's are weakest so if we move up a little bit from that we get and this is maybe the the hardest force for us to visualize or at least the hardest the least intuitive force for me is actually the weak force the weak sometimes called the weak interaction and it's what's responsible for radioactive decay in particular beta minus and beta plus decay and just to give you an example of the actual weak interaction if I had some cesium 137 137 means it has 137 nucleons a nucleon is either a proton or a neutron you add up the protons and neutrons of cesium you get 137 and it is cesium because it has exactly 55 protons now the weak interaction is what's responsible for one of the neutrons essentially one of its corks flipping and turning into a proton and I'm not going to go into detail of what a cork is and all of that and the math can get pretty hairy but I just want to give you an example of what the weak interaction does so if one of these one of these neutrons turns into a proton then we're going to have one extra proton but we have the same number of nucleons instead of a extra Neutron here you now have an extra proton here and so now this is a different atom it is now barium and in that flipping it will actually emit it'll actually emit an electron and an . . .
5	FFF	Which of the following is a difference between gravity and the electromagnetic force?	**[04:34.28–05:52.61]** . . . times the strength of gravity 10 to 36 times the strength of gravity so it kind of puts the weak force in its place it's 10 to the 12 times stronger than the weak force so these are huge numbers that we're talking about either this relative that or even this relative to gravity and so you might be saying well you know the electromagnetic force that's unbelievably strong why doesn't that apply over over these these kind of macro scales like gravity let me write there macro scales macro scales why doesn't it apply to macro scales and actually there's nothing about the electromagnetic force why it can't it or it actually does apply over large distances the reality though is you don't have these huge concentrations of either electric Coulomb charges or magnetism the way you do mass so the mass that you have such huge concentrations it can operate over huge huge distances even though it's way way way weaker than the electromagnetic force the electromagnetic force what happens is because it's both attractive and repulsive it tends to kind of sort itself out so you don't have these huge huge huge concentrations of charge now the other thing you might be wondering about is you know why is it called the electromagnetic force in our everyday life there's things like there's things like the Coulomb force that or the electrostatic force which we're familiar . . .

Supplementary Table 3: (continued)

ID	Question set	Question	Matched lecture transcript text
6	FFF	Electricity and magnetism can be shown to be two cases of the same force if we:	**[04:34.28–05:52.61]** . . . times the strength of gravity 10 to 36 times the strength of gravity so it kind of puts the weak force in its place it's 10 to the 12 times stronger than the weak force so these are huge numbers that we're talking about either this relative that or even this relative to gravity and so you might be saying well you know the electromagnetic force that's unbelievably strong why doesn't that apply over over over these these kind of macro scales like gravity let me write there macro scales macro scales why doesn't it apply to macro scales and actually there's nothing about the electromagnetic force why it can't it or it actually does apply over large distances the reality though is you don't have these huge concentrations of either electric Coulomb charges or magnetism the way you do mass so the mass that you have such huge concentrations it can operate over huge huge distances even though it's way way way weaker than the electromagnetic force the electromagnetic force what happens is because it's both attractive and repulsive it tends to kind of sort itself out so you don't have these huge huge huge concentrations of charge now the other thing you might be wondering about is you know why is it called the electromagnetic force in our everyday life there's things like there's things like the Coulomb force that or the electrostatic force which we're familiar . . .<br><br>**[06:37.31–07:28.1]** . . . that the cool force the electrostatic force and the magnetic force are actually the same thing viewed in different frames of references so I won't go into a lot of detail but just keep that in the back of your mind that they are connected in a future video I'll go more into the intuition of how they are connected and it deals with when what it's more apparent when they're moving when the charges are moving at relativistic frames and you have well I will go to a lot of detail there but just keep in mind that they really are the same force just viewed from different frames of reference now the strongest of the force is probably the best name of them all and that's the strong force that is the strong force the strong force and although you probably haven't seen this yet in our in chemistry classes it actually applies very strongly in . . .
7	FFF	Which of the following are the primary two fundamental forces acting in opposition between the positively-charged protons in an atom's nucleus?	**[00:00.03–01:33.509]** what I want to do in this video is give a very high-level overview of the four fundamental forces four fundamental forces of the universe and I'm going to start with gravity I'm going to start with gravity and it might surprise some of you that gravity is actually the weakest of the four fundamental forces that's surprising because you say wow that's what keeps us glued not glued but it keeps us from jumping off the planet it's what keeps the moon in orbit around the earth the earth in orbit around the Sun the Sun in orbit around the the center of the Milky Way galaxy so it's it's a little bit surprising that it's actually the weakest of the forces and and that starts to make sense when you actually think about things on maybe more of a human scale or a molecular scale or even an atomic scale even on a human scale your computer monitor and you have some type of gravitational attraction but you don't notice it or your cell phone and your wallet there's gravitational attraction but you don't see them being drawn to each other the way you might see two magnets drawn to each other or repelled from each other and if you've got even a smaller scale you'll see that it matters even less we never even talk about gravity and chemistry although the gravity is there but at those scales the other forces really really really start to dominate so gravity's are weakest so if we move up a little bit from that we get and this is maybe the the hardest force for us to visualize or at least the hardest the least intuitive force for me is actually the weak force the weak sometimes called the weak interaction . . .<br><br>**[07:10.13–08:35.24]** . . . reference now the strongest of the force is probably the best name of them all and that's the strong force that is the strong force the strong force and although you probably haven't seen this yet in our in chemistry classes it actually applies very strongly in chemistry because from the get-go when you first learn when you first learn about atoms let me draw a helium atom a helium atom has two protons and it's nucleus two protons in its nucleus and it has two neutrons and then it also has two electrons circulating around so it has an electron and I can draw the electrons is much smaller than well I won't I won't try to do anything in relative size but it has two electrons floating around and one question that may or may not have jumped into your mind when you first saw this model of an atom is like well I see why the electrons are attracted to the nucleus it has a negative Coulomb charge the nucleus has a net positive Coulomb charge but what's not so obvious and that what tends not to sometimes be explained in chemistry class is these two positive charges are sitting right next to each other if the electromagnetic force was the only force in play if the Coulomb force was the only thing happening these guys would just run away from each other they would repel each other and so the only reason why they're able to stick to each other is that there's an even stronger force than the electromagnetic force operating at these very very very small distances . . .
8	FFF	Why does the universe have a very uneven distribution of mass but a relatively equal distribution of charge?	**[08:24.11–10:26.27]** . . . just run away from each other they would repel each other and so the only reason why they're able to stick to each other is that there's an even stronger force than the electromagnetic force operating at these very very very small distances so if you get two of these protons close enough together and the strong force only applies over very very very small distances subatomic I should even say sub nucleic distances then the strong interaction comes into play so then you have the strong interaction actually keeping these charges together and once again just to keep it in in in mind relative to gravity it is 10 to the 38th 10 38 times the strength times the strength of gravity or it's about a hundred times stronger than the electromagnetic force so once again the reason why you don't see the strong force which is the strongest of all of the forces or the weak interaction applying over huge scales is that they really only they only their strength dies off super super fast even when you start going to large radius nucleuses of atoms they strength starts to die off especially for the strong force the reason why you don't see the electromagnetic force operating over large distances even though in theory it can like gravity is that you don't see the type of charge concentrations the way you see mass concentrations in the universe because the charge concentrations tend to sort them out they start to equalize if I have if I have some if I have some positive a huge positive charge there and a huge negative charge there they will attract each other and then become essentially a big lump of neutral charge and once they're a big lump of neutral charge they won't they won't interact with anything else in gravity if you have one mass and another mass and they attract each other then you have another mass that's even better to attracting each other at other masses and so it'll keep attracting things to it so it kind of snowballs the process and that's why gravity can operates on these really really large large objects in our universe in the universe as a whole

Supplementary Table 3: (continued)

ID	Question set	Question	Matched lecture transcript text
9	FFF	In your body, there are a tremendous amount of negatively-charged electrons. Your computer also contains a huge number of negatively-charged electrons. We know that like charges repel, but you and your computer are not repelled apart. Which of the following explains why?	**[00:00.03–01:33.509]** what I want to do in this video is give a very high-level overview of the four fundamental forces four fundamental forces of the universe and I'm going to start with gravity I'm going to start with gravity and it might surprise some of you that gravity is actually the weakest of the four fundamental forces that's surprising because you say wow that's what keeps us glued not glued but it keeps us from jumping off the planet it's what keeps the moon in orbit around the earth the earth in orbit around the Sun the Sun in orbit around the the center of the Milky Way galaxy so it's it's a little bit surprising that it's actually the weakest of the forces and and that starts to make sense when you actually think about things on maybe more of a human scale or a molecular scale or even an atomic scale even on a human scale your computer monitor and you have some type of gravitational attraction but you don't notice it or your cell phone and your wallet there's gravitational attraction but you don't see them being drawn to each other the way you might see two magnets drawn to each other or repelled from each other and if you've got even a smaller scale you'll see that it matters even less we never even talk about gravity and chemistry although the gravity is there but at those scales the other forces really really really start to dominate so gravity's are weakest so if we move up a little bit from that we get and this is maybe the the hardest force for us to visualize or at least the hardest the least intuitive force for me is actually the weak force the weak sometimes called the weak interaction . . .<br><br>**[05:32.6–08:33.14]** . . . force the electromagnetic force what happens is because it's both attractive and repulsive it tends to kind of sort itself out so you don't have these huge huge huge concentrations of charge now the other thing you might be wondering about is you know why is it called the electromagnetic force in our everyday life there's things like there's things like the Coulomb force that or the electrostatic force which we're familiar with positive charges or like charges want to repel if both of these were negative the same thing would be happening and different charges like to attract we've seen this multiple times this is the Coulomb force or the electrostatic force electro static and then on the other side of the word yes you have the magnetic part and magnets you know you have you've played with magnets on your fridge they what's what you know if they're the same side of the magnet they're going to repel each other if they're the opposite sides opposite poles they're going to attract each other so why is it called one force and it's called one force and once again I'm not going to go into detail here it's called one force because it turns out that the cool force the electrostatic force and the magnetic force are actually the same thing viewed in different frames of references so I won't go into a lot of detail but just keep that in the back of your mind that they are connected in a future video I'll go more into the intuition of how they are connected and it deals with when what it's more apparent when they're moving when the charges are moving at relativistic frames and you have well I will go to a lot of detail there but just keep in mind that they really are the same force just viewed from different frames of reference now the strongest of the force is probably the best name of them all and that's the strong force that is the strong force the strong force and although you probably haven't seen this yet in our in chemistry classes it actually applies very strongly in chemistry because from the get-go when you first learn when you first learn about atoms let me draw a helium atom a helium atom has two protons and it's nucleus two protons in its nucleus and it has two neutrons and then it also has two electrons circulating around so it has an electron and I can draw the electrons is much smaller than well I won't I won't try to do anything in relative size but it has two electrons floating around and one question that may or may not have jumped into your mind when you first saw this model of an atom is like well I see why the electrons are attracted to the nucleus it has a negative Coulomb charge the nucleus has a net positive Coulomb charge but what's not so obvious and that what tends not to sometimes be explained in chemistry class is these two positive charges are sitting right next to each other if the electromagnetic force was the only force in play if the Coulomb force was the only thing happening these guys would just run away from each other they would repel each other and so the only reason why they're able to stick to each other is that there's an even stronger force than the electromagnetic force operating . . .
10	FFF	Which of the following is a similarity between the Weak Interaction and the Strong Force?	**[02:52.38–04:43.73]** . . . anti-electron neutrino is these are fundamental particles but this is just what that weak interaction is it's not something that's completely obvious to us it's not kind of this traditional things pulling or pushing away from each other like we would like we associate with the other the other forces now the next strongest force and just to give a sense of how weak gravity is even relative to the weak interaction the weak interaction is 10 to the 25th times the strength of gravity times the strength of gravity and you might anything if this is so strong how come this doesn't operate on planets or us relative to the earth or the reason you know why doesn't this apply to intergalactic distances the way gravity does and the reason is the weak interaction really applies to very small distances very very small distances so it can be much stronger than gravity but only over very very and it really only applies on the sub atomic scale you go anything beyond that it kind of disappears as an actual force as an actual interaction now the next the next force up the hierarchy which is one that we are more familiar with it is something it's what actually dominates most of the chemistry that we deal with and electromagnetism that we deal with and that's the electro magnetic force we write it in magenta electro magnetic magnetic force and just to give a sense this is this is 10 to the 36 times the strength of gravity 10 to 36 times the strength of gravity so it kind of puts the weak force in its place it's 10 to the 12 times stronger than the weak force so these are huge numbers that we're talking about either this . . .
11	FFF	Which force is stronger than the Electromagnetic Force?	**[04:34.28–05:52.61]** . . . times the strength of gravity 10 to 36 times the strength of gravity so it kind of puts the weak force in its place it's 10 to the 12 times stronger than the weak force so these are huge numbers that we're talking about either this relative that or even this relative to gravity and so you might be saying well you know the electromagnetic force that's unbelievably strong why doesn't that apply over over over these these kind of macro scales like gravity let me write there macro scales macro scales why doesn't it apply to macro scales and actually there's nothing about the electromagnetic force why it can't it or it actually does apply over large distances the reality though is you don't have these huge concentrations of either electric Coulomb charges or magnetism the way you do mass so the mass that you have such huge concentrations it can operate over huge huge distances even though it's way way way weaker than the electromagnetic force the electromagnetic force what happens is because it's both attractive and repulsive it tends to kind of sort itself out so you don't have these huge huge huge concentrations of charge now the other thing you might be wondering about is you know why is it called the electromagnetic force in our everyday life there's things like there's things like the Coulomb force that or the electrostatic force which we're familiar . . .
12	FFF	Roughly how many times stronger is the Strong Force than gravity?	**[02:52.38–04:43.73]** . . . anti-electron neutrino is these are fundamental particles but this is just what that weak interaction is it's not something that's completely obvious to us it's not kind of this traditional things pulling or pushing away from each other like we would like we associate with the other the other forces now the next strongest force and just to give a sense of how weak gravity is even relative to the weak interaction the weak interaction is 10 to the 25th times the strength of gravity times the strength of gravity and you might anything if this is so strong how come this doesn't operate on planets or us relative to the earth or the reason you know why doesn't this apply to intergalactic distances the way gravity does and the reason is the weak interaction really applies to very small distances very very small distances so it can be much stronger than gravity but only over very very and it really only applies on the sub atomic scale you go anything beyond that it kind of disappears as an actual force as an actual interaction now the next the next force up the hierarchy which is one that we are more familiar with it is something it's what actually dominates most of the chemistry that we deal with and electromagnetism that we deal with and that's the electro magnetic force we write it in magenta electro magnetic magnetic force and just to give a sense this is this is 10 to the 36 times the strength of gravity 10 to 36 times the strength of gravity so it kind of puts the weak force in its place it's 10 to the 12 times stronger than the weak force so these are huge numbers that we're talking about either this . . .

Supplementary Table 3: (continued)

ID	Question set	Question	Matched lecture transcript text
13	FFF	Which of the following would have to be true for the Weak Interaction to cause repulsion or attraction between two objects?	**[00:00.03–03:02.79]** what I want to do in this video is give a very high-level overview of the four fundamental forces four fundamental forces of the universe and I'm going to start with gravity I'm going to start with gravity and it might surprise some of you that gravity is actually the weakest of the four fundamental forces that's surprising because you say wow that's what keeps us glued not glued but it keeps us from jumping off the planet it's what keeps the moon in orbit around the earth the earth in orbit around the Sun the Sun in orbit around the the center of the Milky Way galaxy so it's it's a little bit surprising that it's actually the weakest of the forces and and that starts to make sense when you actually think about things on maybe more of a human scale or a molecular scale or even an atomic scale even on a human scale your computer monitor and you have some type of gravitational attraction but you don't notice it or your cell phone and your wallet there's gravitational attraction but you don't see them being drawn to each other the way you might see two magnets drawn to each other or repelled from each other and if you've got even a smaller scale you'll see that it matters even less we never even talk about gravity and chemistry although the gravity is there but at those scales the other forces really really really start to dominate so gravity's are weakest so if we move up a little bit from that we get and this is maybe the the hardest force for us to visualize or at least the hardest the least intuitive force for me is actually the weak force the weak sometimes called the weak interaction and it's what's responsible for radioactive decay in particular beta minus and beta plus decay and just to give you an example of the actual weak interaction if I had some cesium 137 137 means it has 137 nucleons a nucleon is either a proton or a neutron you add up the protons and neutrons of cesium you get 137 and it is cesium because it has exactly 55 protons now the weak interaction is what's responsible for one of the neutrons essentially one of its corks flipping and turning into a proton and I'm not going to go into detail of what a cork is and all of that and the math can get pretty hairy but I just want to give you an example of what the weak interaction does so if one of these one of these neutrons turns into a proton then we're going to have one extra proton but we have the same number of nucleons instead of a extra Neutron here you now have an extra proton here and so now this is a different atom it is now barium and in that flipping it will actually emit it'll actually emit an electron and an anti-electron neutrino and I'm not going to go into the details of what an anti-electron neutrino is these are fundamental particles but this is just what that weak interaction is it's not something that's completely obvious to us it's not kind of this traditional things pulling or pushing away from each . . .
14	FFF	Which force keeps us from jumping off of Earth?	**[00:00.03–01:33.509]** what I want to do in this video is give a very high-level overview of the four fundamental forces four fundamental forces of the universe and I'm going to start with gravity I'm going to start with gravity and it might surprise some of you that gravity is actually the weakest of the four fundamental forces that's surprising because you say wow that's what keeps us glued not glued but it keeps us from jumping off the planet it's what keeps the moon in orbit around the earth the earth in orbit around the Sun the Sun in orbit around the the center of the Milky Way galaxy so it's it's a little bit surprising that it's actually the weakest of the forces and and that starts to make sense when you actually think about things on maybe more of a human scale or a molecular scale or even an atomic scale even on a human scale your computer monitor and you have some type of gravitational attraction but you don't notice it or your cell phone and your wallet there's gravitational attraction but you don't see them being drawn to each other the way you might see two magnets drawn to each other or repelled from each other and if you've got even a smaller scale you'll see that it matters even less we never even talk about gravity and chemistry although the gravity is there but at those scales the other forces really really really start to dominate so gravity's are weakest so if we move up a little bit from that we get and this is maybe the the hardest force for us to visualize or at least the hardest the least intuitive force for me is actually the weak force the weak sometimes called the weak interaction . . .
15	FFF	What does the Coulomb Force refer to?	**[05:38.66–06:59.96]** . . . itself out so you don't have these huge huge huge concentrations of charge now the other thing you might be wondering about is you know why is it called the electromagnetic force in our everyday life there's things like there's things like the Coulomb force that or the electrostatic force which we're familiar with positive charges or like charges want to repel if both of these were negative the same thing would be happening and different charges like to attract we've seen this multiple times this is the Coulomb force or the electrostatic force electro static and then on the other side of the word yes you have the magnetic part and magnets you know you have you've played with magnets on your fridge they what's what you know if they're the same side of the magnet they're going to repel each other if they're the opposite sides opposite poles they're going to attract each other so why is it called one force and it's called one force and once again I'm not going to go into detail here it's called one force because it turns out that the cool force the electrostatic force and the magnetic force are actually the same thing viewed in different frames of references so I won't go into a lot of detail but just keep that in the back of your mind that they are connected in a future video I'll go more into the intuition of how they are connected and it deals with when what it's more apparent when they're moving when the charges are moving at relativistic . . .
16	BoS	Which of the following describes the effect of gravity on a cloud of atoms?	**[00:05.67–01:36.71]** . . . and I say huge cloud huge both in distance and in mass if you were to combine all of the hydrogen atoms it would just be this really really massive thing so you have this huge cloud well we know that gravity would make the atoms actually attracted to each other instantly we normally don't think about the gravity of atoms but it would slowly affect these atoms and they'd slowly draw close to each other it would slowly condense they'd slowly they slowly move towards the center of mass of all of the atoms they'd slowly move in and so if we fast forward if we fast forward this cloud is going to get denser and denser it's going to get denser and denser and the hydrogen atoms are going to start bumping into each other and rubbing up against each other and interacting with each other and so it's going to get denser and denser and denser and remember was a huge mass of hydrogen atoms so the temperature is going up the temperature is going up and it'll they'll keep condensing they'll just keep condensing and condensing until something really interesting happens so let's imagine that they've gotten really dense here in the center they've gotten really dense here in the center and there's a bunch of hydrogen atoms all over it's really dense I could never draw the actual number of atoms here this is really give you an idea there's a huge amount of inward pressure from gravity from everything that wants to get to that center of mass of our entire cloud the temperature here the temperature here is approaching 10 million Kelvin and at that point . . .

Supplementary Table 3: (continued)

ID	Question set	Question	Matched lecture transcript text
17	BoS	Which of the following occurs as a cloud of atoms gets more dense?	**[00:05.67–01:36.71]** . . . and I say huge cloud huge both in distance and in mass if you were to combine all of the hydrogen atoms it would just be this really really massive thing so you have this huge cloud well we know that gravity would make the atoms actually attracted to each other instantly we normally don't think about the gravity of atoms but it would slowly affect these atoms and they'd slowly draw close to each other it would slowly condense they'd slowly they slowly move towards the center of mass of all of the atoms they'd slowly move in and so if we fast forward if we fast forward this cloud is going to get denser and denser it's going to get denser and denser and the hydrogen atoms are going to start bumping into each other and rubbing up against each other and interacting with each other and so it's going to get denser and denser and denser and remember was a huge mass of hydrogen atoms so the temperature is going up the temperature is going up and it'll they'll keep condensing they'll just keep condensing and condensing until something really interesting happens so let's imagine that they've gotten really dense here in the center they've gotten really dense here in the center and there's a bunch of hydrogen atoms all over it's really dense I could never draw the actual number of atoms here this is really give you an idea there's a huge amount of inward pressure from gravity from everything that wants to get to that center of mass of our entire cloud the temperature here the temperature here is approaching 10 million Kelvin and at that point . . .<br><br>**[06:50.68–07:52.81]** . . . there's there's questions of well what if what if there just wasn't enough mass to get to this level over here and there actually are things that never get to that quite that threshold to fuse all the way into helium there are a few things that don't quite make the threshold of stars that only fuse to this level so they are generating some of their eat or there are even smaller objects that just get to the point there's a huge temperature and pressure but fusion is not actually occurring inside of the core and something like Jupiter would be an example and you can go several several masses above Jupiter where you get something like that so you have to reach a certain threshold or the mass where the pressure and the temperature due to the heavy mass gets so large that you start this fusion at but the smaller you have above that threshold this the slower the fusion will occur but if you're supermassive the fusion will occur really really fast so that's a general idea of just how stars get formed and why they don't collapse on themselves and why they are these kind of little balls of fusion reactions existing in the universe and the next few videos we'll talk about what happens once that hydrogen fuel and the cure in the core starts to run out . . .
18	BoS	Which temperature does a cloud of hydrogen atoms approach as it gets denser in the process of becoming a star?	**[00:05.67–01:47.82]** . . . and I say huge cloud huge both in distance and in mass if you were to combine all of the hydrogen atoms it would just be this really really massive thing so you have this huge cloud well we know that gravity would make the atoms actually attracted to each other instantly we normally don't think about the gravity of atoms but it would slowly affect these atoms and they'd slowly draw close to each other it would slowly condense they'd slowly they slowly move towards the center of mass of all of the atoms they'd slowly move in and so if we fast forward if we fast forward this cloud is going to get denser and denser it's going to get denser and denser and the hydrogen atoms are going to start bumping into each other and rubbing up against each other and interacting with each other and so it's going to get denser and denser and denser and remember was a huge mass of hydrogen atoms so the temperature is going up the temperature is going up and it'll they'll keep condensing they'll just keep condensing and condensing until something really interesting happens so let's imagine that they've gotten really dense here in the center they've gotten really dense here in the center and there's a bunch of hydrogen atoms all over it's really dense I could never draw the actual number of atoms here this is really give you an idea there's a huge amount of inward pressure from gravity from everything that wants to get to that center of mass of our entire cloud the temperature here the temperature here is approaching 10 million Kelvin and at that point something neat happens it to kind of realize the neat thing that's happening let's remember what a hydrogen atom looks like a hydrogen and even more I'm just going to focus on the hydrogen . . .<br><br>**[05:50.32–07:00.25]** . . . helium and by definition helium has two protons and two neutrons so it has or we're talking about helium four in particular that isotope of helium it has an atomic mass of four and the whole this process releases a ton of energy because the atomic mass of the helium that gets produced is slightly lower than four of its of the atomic four times the atomic mass of each of the Constituent hydrogen's so all of this energy all of this energy from the fusion but it needs super high pressure super high temperatures to happen keeps keeps the star from collapsing and once the star is in in this stage once it is once it is using hydrogen it is fusing hydrogen in its core where the pressure and the temperatures of most to form helium it is now in its main sequence this is now a main sequence star main main sequence star and that's actually where the Sun is right now now there's there's questions of well what if what if there just wasn't enough mass to get to this level over here and there actually are things that never get to that quite that threshold to fuse all the way into helium there are a few . . .
19	BoS	Which of the following can overcome the Coulomb Force?	**[02:24.67–03:53.08]** . . . learned about the four force is that if they did get close enough to each other that if they did get if somehow they're under huge temperatures and huge pressures you were able to get these two protons close enough to each other then all of a sudden the strong force will overtake it's much stronger than the Coulomb force and that these two hydrogen's will actually these this these nucleuses would actually fuse or that nuclei well anyway they would actually fuse together and so that is what actually happens once this gets hot and dense enough you now have enough pressure enough temperature to overcome the Coulomb force and bring these protons close enough to each other for fusion to occur for fusion fusion ignition ignition and the reason why is and I want to be very careful it's not ignition it's not combustion in the traditional sense it's not like you're burning a carbon molecule with out in the presence of oxygen it's not combustion it's ignition and the reason why it's called ignition is because when two of these protons or two of the nucleus is fuse the resulting the resulting nucleus has a slightly smaller mass and so in the first stage of this you actually have you actually have two protons two protons under enough pressure obviously this would not happen with just the Coulomb forces at with enough pressure to get close enough and then the strong interaction actually actually keeps them together one of these guys degrades in a neutron degrades into a neutron and the . . .
20	BoS	Which of the following prevents a star from collapsing as a result of gravity?	**[03:36.34–05:04.75]** . . . you actually have you actually have two protons two protons under enough pressure obviously this would not happen with just the Coulomb forces at with enough pressure to get close enough and then the strong interaction actually actually keeps them together one of these guys degrades in a neutron degrades into a neutron and the resulting mass of the combined protons is lower than the mass of each of the original by a little bit but that a little bit of mass results in a lot of energy plus plus energy and this energy is why we call it ignition and so what this energy does is it provides a little bit of outward pressure so that this thing doesn't keep keep collapsing so once you get pressure enough the fusion occurs and then that energy provides outward pressure to what is now a star what is now a star so now we are at where we actually have the ignition at the center we have and we still have all of the other molecules trying to get in providing the pressure for this fusion ignition now what is the hydrogen being fused into well on the first step of the first step of the reaction and I'm just kind of doing the most basic type of fusion that exhibit ours the hydrogen gets fused into deuterium deuterium of trouble spelling which is another way of calling heavy hydrogen this is still hydrogen because it has one proton and one Neutron now it is not . . .

Supplementary Table 3: (continued)

ID	Question set	Question	Matched lecture transcript text
21	BoS	How are supermassive stars different from other stars?	**[06:48.19–07:52.81]** . . . actually where the Sun is right now now there's there's questions of well what if what if there just wasn't enough mass to get to this level over here and there actually are things that never get to that quite that threshold to fuse all the way into helium there are a few things that don't quite make the threshold of stars that only fuse to this level so they are generating some of their eat or there are even smaller objects that just get to the point there's a huge temperature and pressure but fusion is not actually occurring inside of the core and something like Jupiter would be an example and you can go several several masses above Jupiter where you get something like that so you have to reach a certain threshold or the mass where the pressure and the temperature due to the heavy mass gets so large that you start this fusion at but the smaller you have above that threshold this the slower the fusion will occur but if you're supermassive the fusion will occur really really fast so that's a general idea of just how stars get formed and why they don't collapse on themselves and why they are these kind of little balls of fusion reactions existing in the universe and the next few videos we'll talk about what happens once that hydrogen fuel and the cure in the core starts to run out . . .
22	BoS	Which of the following is the FIRST product of two hydrogen atoms fusing together?	**[00:05.67–01:36.71]** . . . and I say huge cloud huge both in distance and in mass if you were to combine all of the hydrogen atoms it would just be this really really massive thing so you have this huge cloud well we know that gravity would make the atoms actually attracted to each other instantly we normally don't think about the gravity of atoms but it would slowly affect these atoms and they'd slowly draw close to each other it would slowly condense they'd slowly they slowly move towards the center of mass of all of the atoms they'd slowly move in and so if we fast forward if we fast forward this cloud is going to get denser and denser it's going to get denser and denser and the hydrogen atoms are going to start bumping into each other and rubbing up against each other and interacting with each other and so it's going to get denser and denser and denser and remember was a huge mass of hydrogen atoms so the temperature is going up the temperature is going up and it'll they'll keep condensing they'll just keep condensing and condensing until something really interesting happens so let's imagine that they've gotten really dense here in the center they've gotten really dense here in the center and there's a bunch of hydrogen atoms all over it's really dense I could never draw the actual number of atoms here this is really give you an idea there's a huge amount of inward pressure from gravity from everything that wants to get to that center of mass of our entire cloud the temperature here the temperature here is approaching 10 million Kelvin and at that point . . .<br><br>**[02:24.67–03:53.08]** . . . learned about the four force is that if they did get close enough to each other that if they did get if somehow they're under huge temperatures and huge pressures you were able to get these two protons close enough to each other then all of a sudden the strong force will overtake it's much stronger than the Coulomb force and that these two hydrogen's will actually these this these nucleuses would actually fuse or that nuclei well anyway they would actually fuse together and so that is what actually happens once this gets hot and dense enough you now have enough pressure enough temperature to overcome the Coulomb force and bring these protons close enough to each other for fusion to occur for fusion fusion ignition ignition and the reason why is and I want to be very careful it's not ignition it's not combustion in the traditional sense it's not like you're burning a carbon molecule with out in the presence of oxygen it's not combustion it's ignition and the reason why it's called ignition is because when two of these protons or two of the nucleus is fuse the resulting the resulting nucleus has a slightly smaller mass and so in the first stage of this you actually have you actually have two protons two protons under enough pressure obviously this would not happen with just the Coulomb forces at with enough pressure to get close enough and then the strong interaction actually actually keeps them together one of these guys degrades in a neutron degrades into a neutron and the . . .
23	BoS	Once hydrogen atoms get close enough together, which of the following keeps them together?	**[00:05.67–01:36.71]** . . . and I say huge cloud huge both in distance and in mass if you were to combine all of the hydrogen atoms it would just be this really really massive thing so you have this huge cloud well we know that gravity would make the atoms actually attracted to each other instantly we normally don't think about the gravity of atoms but it would slowly affect these atoms and they'd slowly draw close to each other it would slowly condense they'd slowly they slowly move towards the center of mass of all of the atoms they'd slowly move in and so if we fast forward if we fast forward this cloud is going to get denser and denser it's going to get denser and denser and the hydrogen atoms are going to start bumping into each other and rubbing up against each other and interacting with each other and so it's going to get denser and denser and denser and remember was a huge mass of hydrogen atoms so the temperature is going up the temperature is going up and it'll they'll keep condensing they'll just keep condensing and condensing until something really interesting happens so let's imagine that they've gotten really dense here in the center they've gotten really dense here in the center and there's a bunch of hydrogen atoms all over it's really dense I could never draw the actual number of atoms here this is really give you an idea there's a huge amount of inward pressure from gravity from everything that wants to get to that center of mass of our entire cloud the temperature here the temperature here is approaching 10 million Kelvin and at that point . . .<br><br>**[02:24.67–03:53.08]** . . . learned about the four force is that if they did get close enough to each other that if they did get if somehow they're under huge temperatures and huge pressures you were able to get these two protons close enough to each other then all of a sudden the strong force will overtake it's much stronger than the Coulomb force and that these two hydrogen's will actually these this these nucleuses would actually fuse or that nuclei well anyway they would actually fuse together and so that is what actually happens once this gets hot and dense enough you now have enough pressure enough temperature to overcome the Coulomb force and bring these protons close enough to each other for fusion to occur for fusion fusion ignition ignition and the reason why is and I want to be very careful it's not ignition it's not combustion in the traditional sense it's not like you're burning a carbon molecule with out in the presence of oxygen it's not combustion it's ignition and the reason why it's called ignition is because when two of these protons or two of the nucleus is fuse the resulting the resulting nucleus has a slightly smaller mass and so in the first stage of this you actually have you actually have two protons two protons under enough pressure obviously this would not happen with just the Coulomb forces at with enough pressure to get close enough and then the strong interaction actually actually keeps them together one of these guys degrades in a neutron degrades into a neutron and the . . .
24	BoS	When two nuclei fuse together, how does the mass of the combined nucleus compare to the mass of each of the original nucleus?	**[02:26.05–04:49.15]** . . . they did get close enough to each other that if they did get if somehow they're under huge temperatures and huge pressures you were able to get these two protons close enough to each other then all of a sudden the strong force will overtake it's much stronger than the Coulomb force and that these two hydrogen's will actually these this these nucleuses would actually fuse or that nuclei well anyway they would actually fuse together and so that is what actually happens once this gets hot and dense enough you now have enough pressure enough temperature to overcome the Coulomb force and bring these protons close enough to each other for fusion to occur for fusion fusion ignition ignition and the reason why is and I want to be very careful it's not ignition it's not combustion in the traditional sense it's not like you're burning a carbon molecule with out in the presence of oxygen it's not combustion it's ignition and the reason why it's called ignition is because when two of these protons or two of the nucleus is fuse the resulting the resulting nucleus has a slightly smaller mass and so in the first stage of this you actually have you actually have two protons two protons under enough pressure obviously this would not happen with just the Coulomb forces at with enough pressure to get close enough and then the strong interaction actually actually keeps them together one of these guys degrades in a neutron degrades into a neutron and the resulting mass of the combined protons is lower than the mass of each of the original by a little bit but that a little bit of mass results in a lot of energy plus plus energy and this energy is why we call it ignition and so what this energy does is it provides a little bit of outward pressure so that this thing doesn't keep keep collapsing so once you get pressure enough the fusion occurs and then that energy provides outward pressure to what is now a star what is now a star so now we are at where we actually have the ignition at the center we have and we still have all of the other molecules trying to get in providing the pressure for this fusion ignition now what is the hydrogen being fused into well on the first step of the first step of the reaction and I'm just kind of doing the . . .

Supplementary Table 3: (continued)

ID	Question set	Question	Matched lecture transcript text
25	BoS	If we say that our Sun is a main sequence star, what does that tell us about the Sun?	**[05:47.08–07:01.75]** . . . eventually gets fused and I'm not going to the detail of the reaction into helium and by definition helium has two protons and two neutrons so it has or we're talking about helium four in particular that isotope of helium it has an atomic mass of four and the whole this process releases a ton of energy because the atomic mass of the helium that gets produced is slightly lower than four of its of the atomic four times the atomic mass of each of the Constituent hydrogen's so all of this energy all of this energy from the fusion but it needs super high pressure super high temperatures to happen keeps keeps the star from collapsing and once the star is in in this stage once it is once it is using hydrogen it is fusing hydrogen in its core where the pressure and the temperatures of most to form helium it is now in its main sequence this is now a main sequence star main main sequence star and that's actually where the Sun is right now now there's there's questions of well what if what if there just wasn't enough mass to get to this level over here and there actually are things that never get to that quite that threshold to fuse all the way into helium there are a few things that don't quite make the . . .
26	BoS	Which force would cause a massive cloud of hydrogen atoms to move together?	**[00:05.67–01:36.71]** . . . and I say huge cloud huge both in distance and in mass if you were to combine all of the hydrogen atoms it would just be this really really massive thing so you have this huge cloud well we know that gravity would make the atoms actually attracted to each other instantly we normally don't think about the gravity of atoms but it would slowly affect these atoms and they'd slowly draw close to each other it would slowly condense they'd slowly they slowly move towards the center of mass of all of the atoms they'd slowly move in and so if we fast forward if we fast forward this cloud is going to get denser and denser it's going to get denser and denser and the hydrogen atoms are going to start bumping into each other and rubbing up against each other and interacting with each other and so it's going to get denser and denser and denser and remember was a huge mass of hydrogen atoms so the temperature is going up the temperature is going up and it'll they'll keep condensing they'll just keep condensing and condensing until something really interesting happens so let's imagine that they've gotten really dense here in the center they've gotten really dense here in the center and there's a bunch of hydrogen atoms all over it's really dense I could never draw the actual number of atoms here this is really give you an idea there's a huge amount of inward pressure from gravity from everything that wants to get to that center of mass of our entire cloud the temperature here the temperature here is approaching 10 million Kelvin and at that point . . .<br><br>**[02:26.05–03:51.37]** . . . they did get close enough to each other that if they did get if somehow they're under huge temperatures and huge pressures you were able to get these two protons close enough to each other then all of a sudden the strong force will overtake it's much stronger than the Coulomb force and that these two hydrogen's will actually these this these nucleuses would actually fuse or that nuclei well anyway they would actually fuse together and so that is what actually happens once this gets hot and dense enough you now have enough pressure enough temperature to overcome the Coulomb force and bring these protons close enough to each other for fusion to occur for fusion fusion ignition ignition and the reason why is and I want to be very careful it's not ignition it's not combustion in the traditional sense it's not like you're burning a carbon molecule with out in the presence of oxygen it's not combustion it's ignition and the reason why it's called ignition is because when two of these protons or two of the nucleus is fuse the resulting the resulting nucleus has a slightly smaller mass and so in the first stage of this you actually have you actually have two protons two protons under enough pressure obviously this would not happen with just the Coulomb forces at with enough pressure to get close enough and then the strong interaction actually actually keeps them together one of these guys degrades in a neutron . . .
27	BoS	Which of the following occurs as density increases?	**[06:48.19–07:52.81]** . . . actually where the Sun is right now now there's there's questions of well what if what if there just wasn't enough mass to get to this level over here and there actually are things that never get to that quite that threshold to fuse all the way into helium there are a few things that don't quite make the threshold of stars that only fuse to this level so they are generating some of their eat or there are even smaller objects that just get to the point there's a huge temperature and pressure but fusion is not actually occurring inside of the core and something like Jupiter would be an example and you can go several several masses above Jupiter where you get something like that so you have to reach a certain threshold or the mass where the pressure and the temperature due to the heavy mass gets so large that you start this fusion at but the smaller you have above that threshold this the slower the fusion will occur but if you're supermassive the fusion will occur really really fast so that's a general idea of just how stars get formed and why they don't collapse on themselves and why they are these kind of little balls of fusion reactions existing in the universe and the next few videos we'll talk about what happens once that hydrogen fuel and the cure in the core starts to run out . . .
28	BoS	Which of the following is a product of Hydrogen fusion?	**[06:48.19–07:52.81]** . . . actually where the Sun is right now now there's there's questions of well what if what if there just wasn't enough mass to get to this level over here and there actually are things that never get to that quite that threshold to fuse all the way into helium there are a few things that don't quite make the threshold of stars that only fuse to this level so they are generating some of their eat or there are even smaller objects that just get to the point there's a huge temperature and pressure but fusion is not actually occurring inside of the core and something like Jupiter would be an example and you can go several several masses above Jupiter where you get something like that so you have to reach a certain threshold or the mass where the pressure and the temperature due to the heavy mass gets so large that you start this fusion at but the smaller you have above that threshold this the slower the fusion will occur but if you're supermassive the fusion will occur really really fast so that's a general idea of just how stars get formed and why they don't collapse on themselves and why they are these kind of little balls of fusion reactions existing in the universe and the next few videos we'll talk about what happens once that hydrogen fuel and the cure in the core starts to run out . . .
29	BoS	Which of the following terms accurately describes the Sun?	
30	BoS	Which of the following terms best describes a fusion reaction?	**[04:51.639–06:05.83]** . . . most basic type of fusion that exhibit ours the hydrogen gets fused into deuterium deuterium of trouble spelling which is another way of calling heavy hydrogen this is still hydrogen because it has one proton and one Neutron now it is not helium yet it does not have two it does not have two protons but then the deuterium keeps fusing and then we eventually end up with and then we end up with helium and we can even see that on the periodic table I lost my periodic table well I'll show you in the next video but we know hydrogen hydrogen hydrogen in its atomic state has an atomic number of one and it also has a mass of one it only has one nucleon and it's nucleus but it's being fused it goes to hydrogen too which is deuterium which is one Neutron one proton in its nucleus two nucleons and then that eventually gets fused and I'm not going to the detail of the reaction into helium and by definition helium has two protons and two neutrons so it has or we're talking about helium four in particular that isotope of helium it has an atomic mass of four and the whole this process releases a ton of energy because the atomic mass of the helium . . .<br><br>**[06:50.68–07:52.81]** . . . there's there's questions of well what if what if there just wasn't enough mass to get to this level over here and there actually are things that never get to that quite that threshold to fuse all the way into helium there are a few things that don't quite make the threshold of stars that only fuse to this level so they are generating some of their eat or there are even smaller objects that just get to the point there's a huge temperature and pressure but fusion is not actually occurring inside of the core and something like Jupiter would be an example and you can go several several masses above Jupiter where you get something like that so you have to reach a certain threshold or the mass where the pressure and the temperature due to the heavy mass gets so large that you start this fusion at but the smaller you have above that threshold this the slower the fusion will occur but if you're supermassive the fusion will occur really really fast so that's a general idea of just how stars get formed and why they don't collapse on themselves and why they are these kind of little balls of fusion reactions existing in the universe and the next few videos we'll talk about what happens once that hydrogen fuel and the cure in the core starts to run out . . .

Supplementary figures

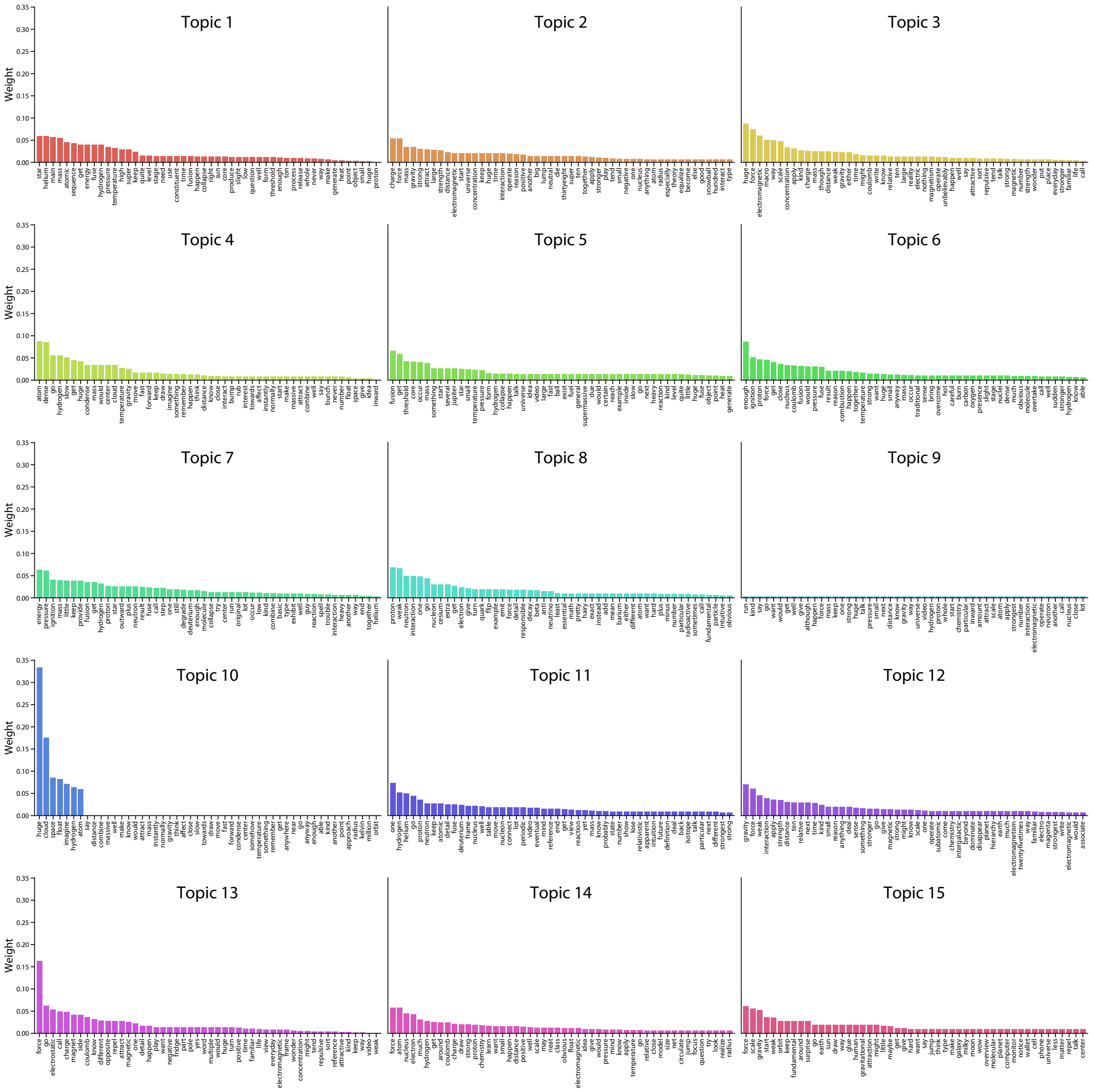

Supplementary Figure 1: Topic-word distributions. Each topic is defined by a distribution of weights over words in the topic model's vocabulary (i.e., the union of all unique words from the two lectures' transcripts, excluding stop-words, after preprocessing). Each plot above displays the weights for the 50 words weighted most heavily by an individual topic extracted from the lectures' contents (see *Constructing text embeddings of multiple lectures and questions*).

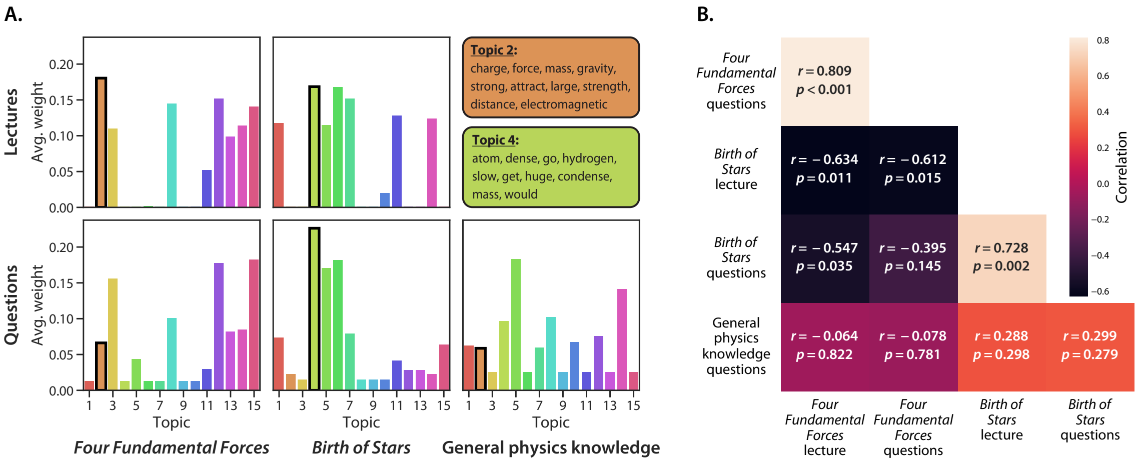

Supplementary Figure 2: Topic weights. A. Average topic weights for each lecture and question set. The bar plots display each topic's average weight across lecture timepoints (top row) and questions (bottom row); colors denote topics. The top-weighted words from the highest-weighted topic from each lecture are displayed in the upper right (orange: topic 2; yellow-green: topic 4). The top-weighted words from the full set of topics may be found in Supplementary Table 2. **B. Relationships between average topic weights.** Pairwise correlations between the distributions of average topic weights for each lecture and question set. Each row and column corresponds to a bar plot in Panel A. Also see Figure 3 in the main text.

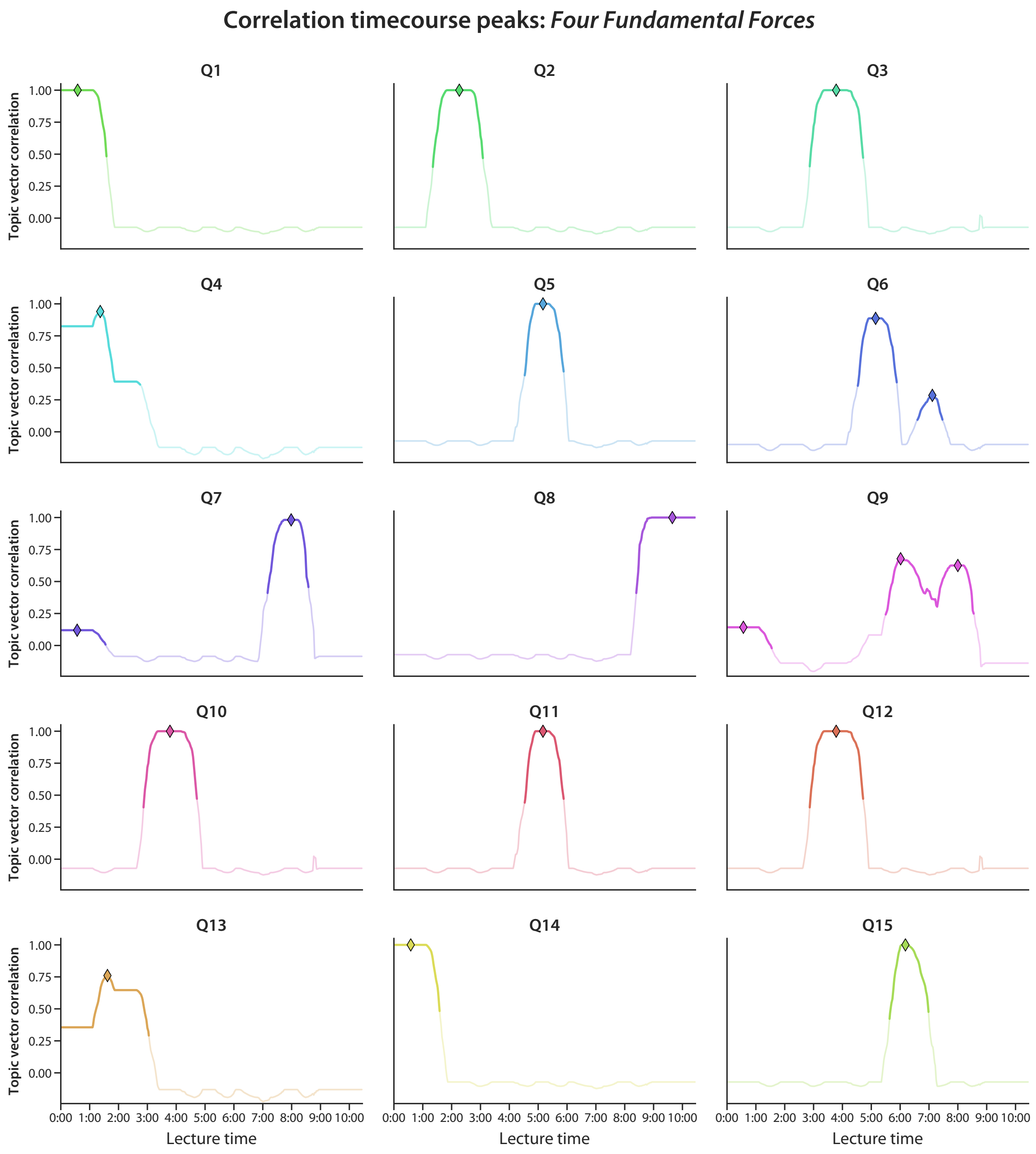

Supplementary Figure 3: Topic correlation time series plots for individual *Four Fundamental Forces* questions. Each panel displays a time series plot showing how the question's topic vector correlates with the topic vectors for each timepoint of *Four Fundamental Forces*. The shaded regions in each curve denote the full width at half maxima (FWHM) of the peak(s) in the curve. These FWHM intervals were used to identify the corresponding lecture intervals displayed in Supplementary Table 3. The diamonds denote the peak correlation values (local maxima) within each shaded region.

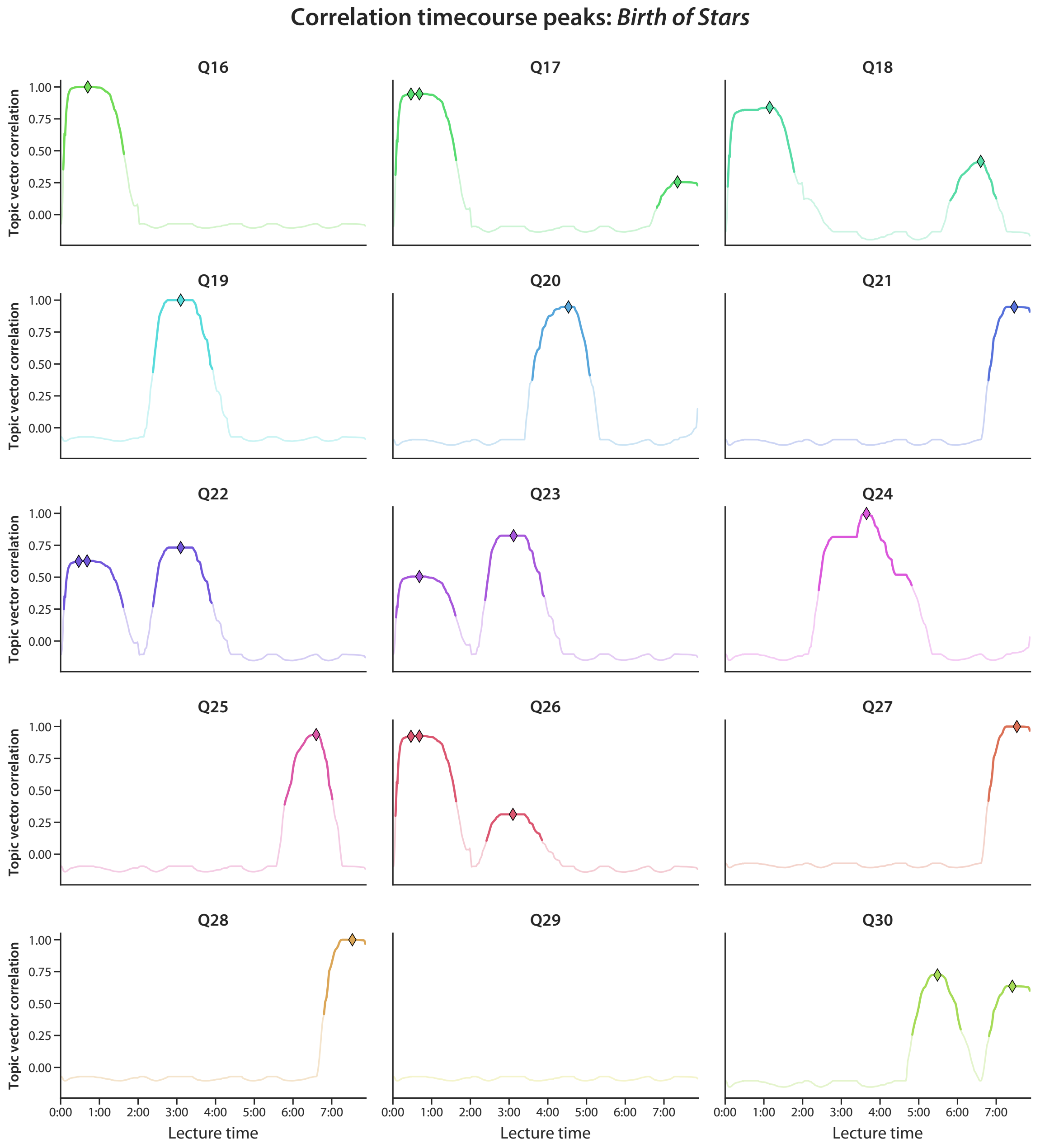

Supplementary Figure 4: Topic correlation time series plots for individual *Birth of Stars* questions. Each panel displays a time series plot showing how the question's topic vector correlates with the topic vectors for each timepoint of *Birth of Stars*. The shaded regions in each curve denote the full width at half maxima (FWHM) of the peak(s) in the curve. These FWHM intervals were used to identify the corresponding lecture intervals displayed in Supplementary Table 3. The diamonds denote the peak correlation values (local maxima) within each shaded region.

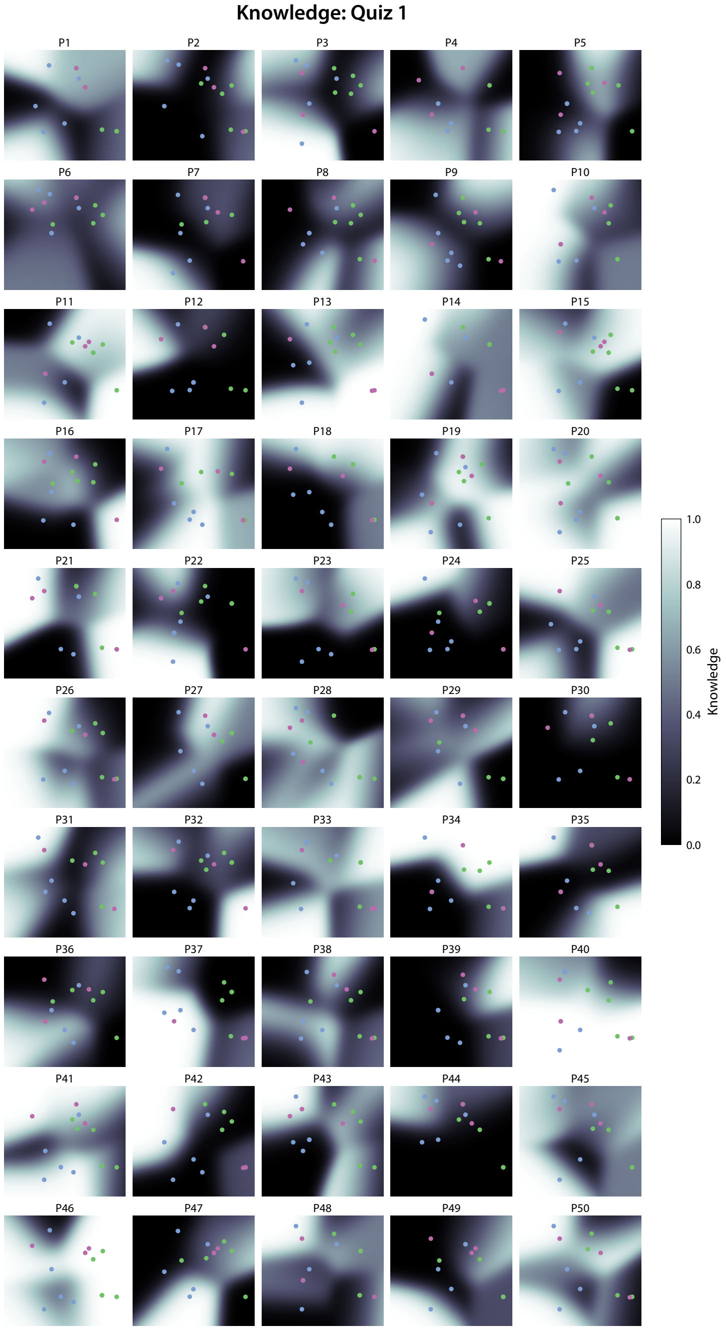

Supplementary Figure 5: Individual participants' knowledge maps estimated from Quiz 1 responses. Each panel is in the same format as the knowledge map displayed in the left panel of Figure 7A in the main text, but here each participant's map shows only the questions they answered on Quiz 1.

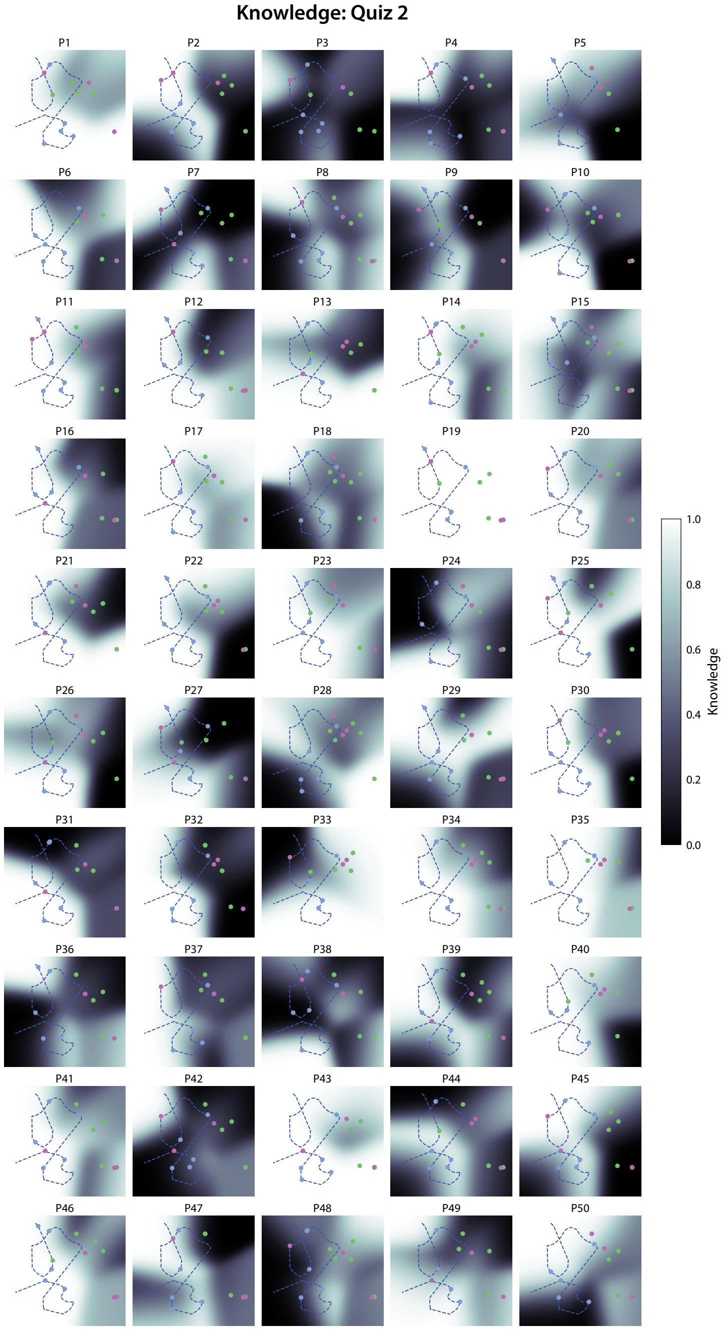

Supplementary Figure 6: Individual participants' knowledge maps estimated from Quiz 2 responses. Each panel is in the same format as the knowledge map displayed in the middle panel of Figure 7A in the main text, but here each participant's map shows only the questions they answered on Quiz 2.

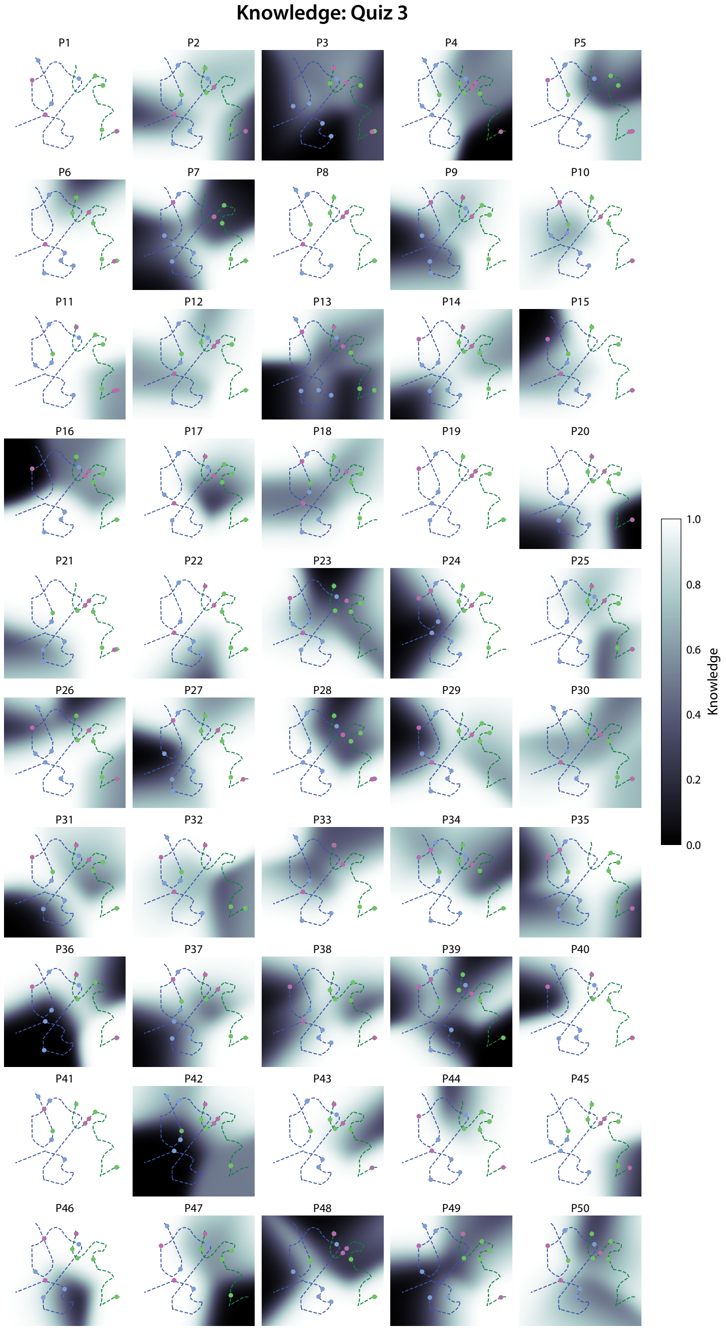

Supplementary Figure 7: Individual participants' knowledge maps estimated from Quiz 3 responses. Each panel is in the same format as the knowledge map displayed in the right panel of Figure 7A in the main text, but here each participant's map shows only the questions they answered on Quiz 3.

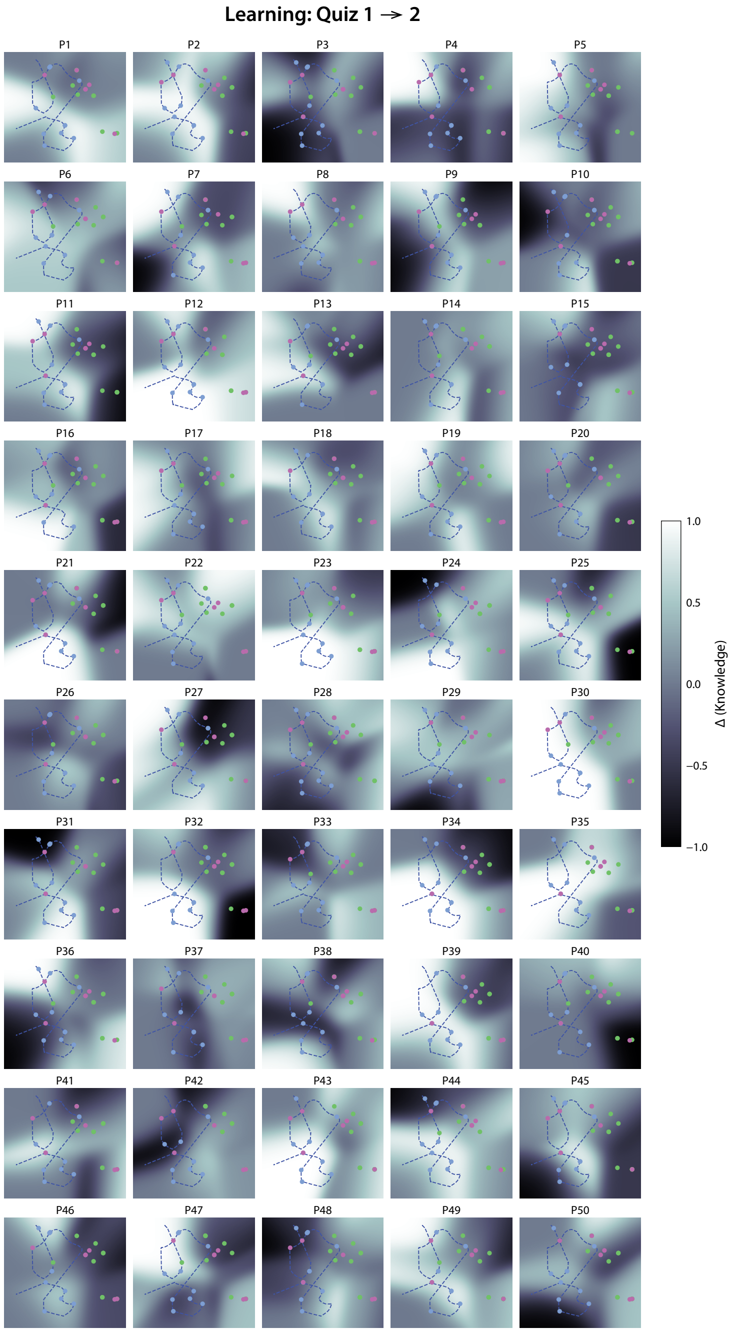

Supplementary Figure 8: Individual participants' learning maps estimated from Quiz 1 and 2 responses. Each panel is in the same format as the learning map displayed in the left panel of Figure 7B in the main text, but here each participant's map shows only the questions they answered on Quizzes 1 & 2.

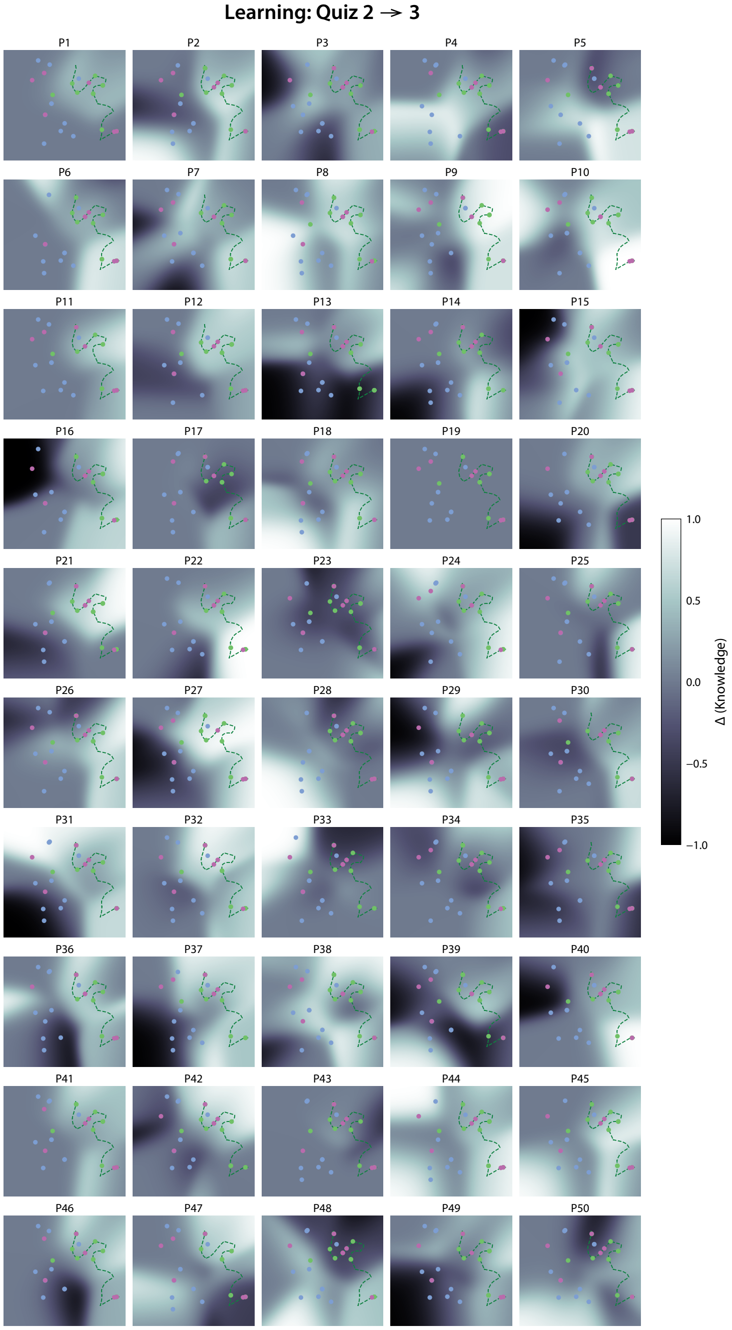

Supplementary Figure 9: Individual participants' learning maps estimated from Quiz 2 and 3 responses. Each panel is in the same format as the learning map displayed in the right panel of Figure 7B in the main text, but here each participant's map shows only the questions they answered on Quizzes 2 & 3.

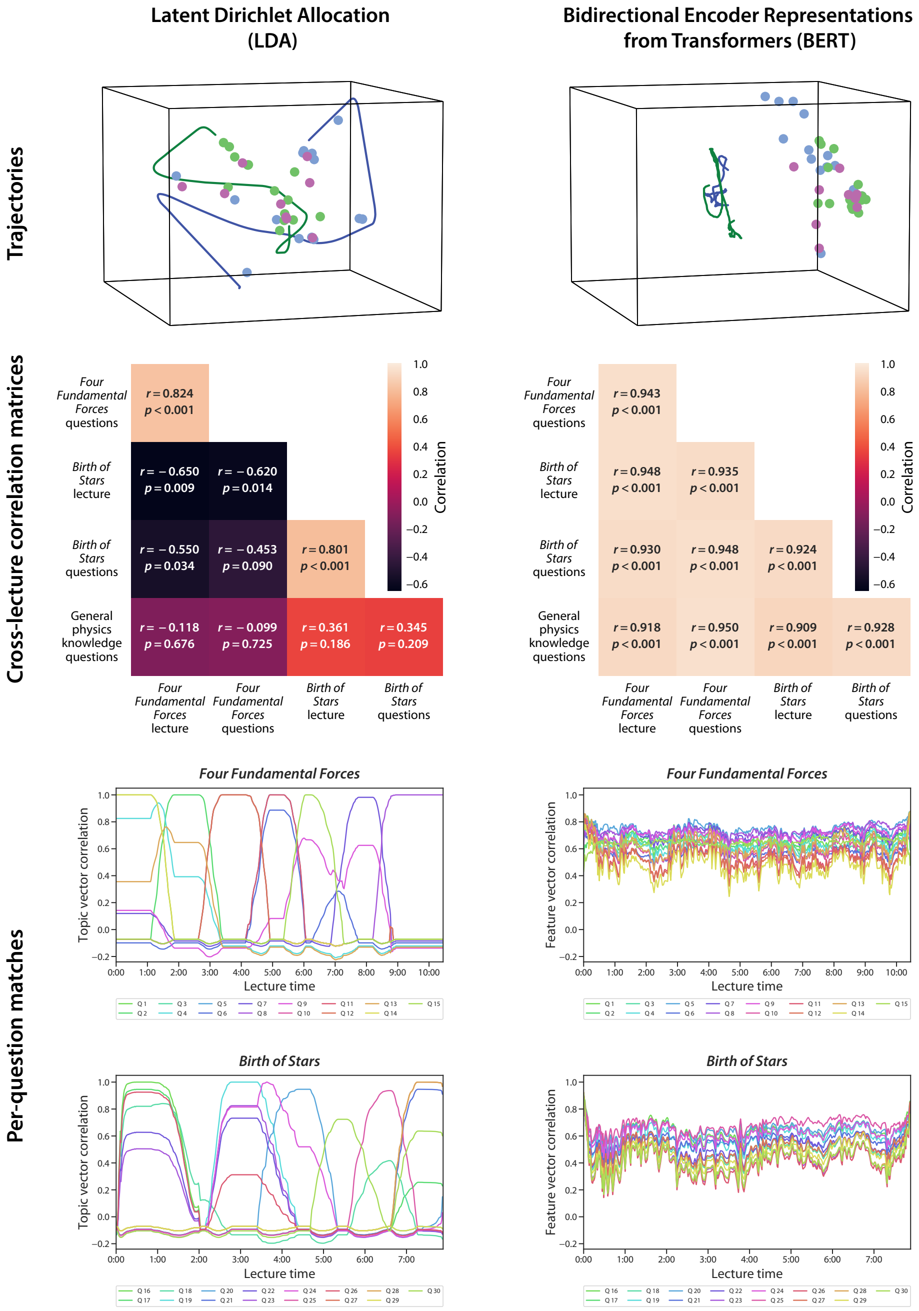

Supplementary Figure 10: Comparing LDA vs. BERT embeddings. The rows display the equivalents of main text Figure 2C (top row), Figure 3B (second row), and Figures 4A and 4B (bottom two rows) computed using LDA (left) and BERT (right).

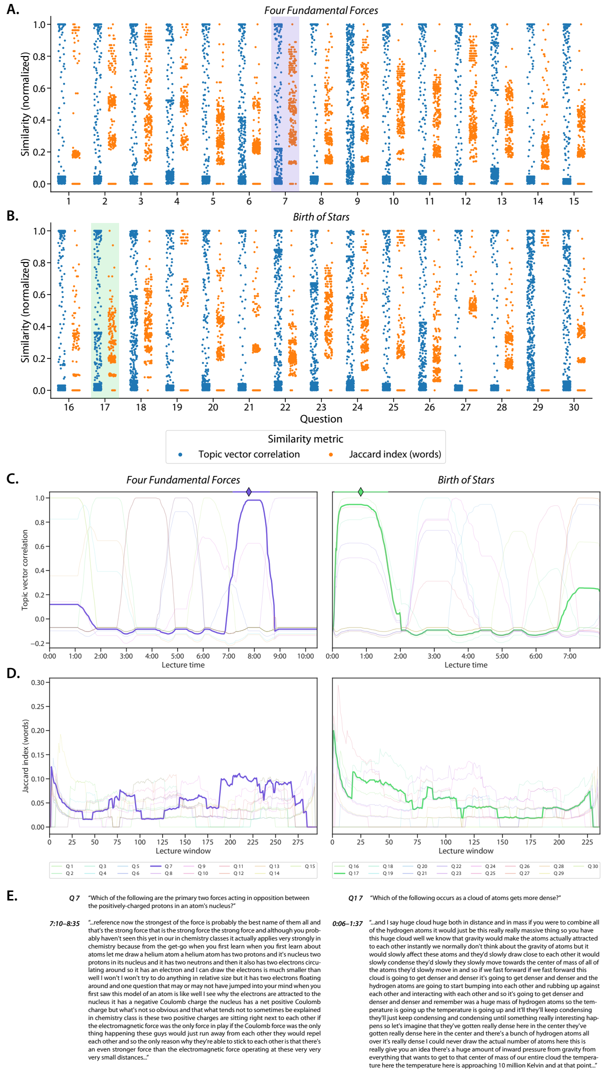

Supplementary Figure 11: Topic correlations versus word overlap. A. Single-timepoint comparisons for *Four Fundamental Forces*. Each question about *Four Fundamental Forces* was compared to each timepoint of the lecture (dots). The blue dots denote the correlations between the topic vectors, and the orange dots denote Jaccard similarities (defined as the number of unique words in common between the question and corresponding window of the lecture, divided by the total number of unique words in both). **B. Single-timepoint comparisons for *Birth of Stars*.** Same format as Panel A. **C. Topic correlation time series plots.** This panel is in the same format as Figure 4A from the main text, but here the example question highlighted in purple in Panel A has been emphasized for the *Four Fundamental Forces* traces on the left, and the example question highlighted in green in Panel B has been emphasized for the *Birth of Stars* traces on the right. **D. Jaccard similarity time series plots.** These panels are in the same format as Panel C, but they display the Jaccard similarity time series for the same questions. **E. Question and lecture text.** The text for the example purple (left) and green (right) questions is shown on top, and the text from the best-matching intervals of the lectures (computed using topic correlations; see Supp. Tab. 3) are shown on the bottom.

Supplementary discussion

In our study, we relate the conceptual contents of course lectures and quiz questions by their coordinates in a high-dimensional feature space, which we construct using a relatively simple text embedding model (Latent Dirichlet Allocation; 1) trained on overlapping sliding windows of text from the lectures' transcripts (see *Constructing text embeddings of multiple lectures and questions*). In comparing our approach to various alternative methods of characterizing the lectures' and questions' contents, we found that embeddings derived from our corpus-specific LDA model exhibited a number of desirable properties that both simpler and more complex alternatives did not. For example, we found that analogous embeddings derived from more modern and complex models trained on much larger and more diverse text corpora (e.g., BERT, 2; GPT, 4; LLaMa, 3) tended to afford poor resolution within the content space spanned by individual course lectures and quiz questions. To illustrate this contrast, in Supplementary Figure 10 we display a subset of visualizations from the main text based on our LDA embeddings (left column) alongside analogous visualizations based instead on BERT embeddings (right column).

The top row of Supplementary Figure 10 displays PCA projections of the two lectures' trajectories and 39 quiz questions' coordinates within the embedding space of each model (analogous to Figure 2C in the main text). Qualitatively comparing these two sets of projections reveals a number of notable differences. For example, whereas the LDA embeddings of the lectures and questions are "near" each other (i.e., the convex hull enclosing the two lectures' trajectories is highly overlapping with the convex hull enclosing the questions' embeddings), the BERT embeddings of the lectures and questions are instead largely distinct. The LDA embeddings of the questions about each lecture and the corresponding lecture's trajectory are also similar: *Four Fundamental Forces*-related questions (blue dots) generally appear closer to the *Four Fundamental Forces* lecture trajectory (blue line), while *Birth of Stars*-related questions (green dots) generally appear closer to the *Birth of Stars* lecture trajectory (green line). The BERT embeddings of the lectures and questions do not display this property.

The second row of Supplementary Figure 10 offers a more quantitative version of the compar-

isons above. Here, for the embeddings derived from each model, we show the pairwise correlations between the variance in each feature dimension's weight, across timepoints from each lecture and quiz questions about each content area (analogous to main text Fig. 3B). The LDA embeddings show a strong contrast between same-content embeddings and across-content embeddings. In other words, the the amount of "information" about different features (i.e., topics) reflected in each lecture's content is highly similar to the amount of information reflected in the contents of its corresponding quiz questions, and dissimilar from that of other subsets of quiz questions. The BERT embeddings, by contrast, do not exhibit these clear distinctions between content areas, and instead represent *all* course materials used in our study highly similarly overall. This indicates that, relative to enormous breadth of conceptual content BERT embeddings can represent, representations of concepts that are specifically related to *Four Fundamental Forces*, specifically related to *Birth of Stars*, and related to physics more generally are so similar as to be almost indistinguishable. Additionally, the relative similarities (i.e., correlations) between lectures and question sets indicate that both lectures are represented more similarly to each other than to any subset of quiz questions, and each subset of quiz questions is represented more similarly to other quiz questions than to either lecture. This mirrors the separation between the lectures and questions visible in their PCA projections above, and suggests that BERT's representations of the subtle, specific semantic features that link the lecture and questions within each content area, and distinguish between different content areas, are sufficiently weak that they are overpowered by representations of *syntactic* features that tend to distinguish questions from lectures more broadly (e.g., beginning with words like "What", "Which", or "Why", ending with a question mark, etc.). Taken together, first two rows of Supplementary Figure 10 suggest that a simple LDA model trained on the specific lectures used in our study can "match" quiz questions to their corresponding lectures by similarities in their embedding weights, while BERT (trained on a much broader text corpus) cannot.

We also examined both models' abilities to match the quiz questions to specific temporal intervals within the lectures. The third and fourth rows of Supplementary Figure 10 display the time series of correlations between the embedding weights for each quiz question and each timepoint of its corresponding lecture (third row: *Four Fundamental Forces*; fourth row: *Birth of Stars*;

analogous to main text Fig. 4). When computed using LDA embeddings, the questions' correlation time series generally appear fairly different from each other (i.e., individual questions' traces can be easily visually separated), whereas their correlation time series computed using BERT embeddings are all highly similar. This indicates that the LDA embeddings are capturing some differences in content between the questions about a given lecture that the BERT embeddings are not. The time series plots of individual questions' correlations also exhibit clear "peaks" when computed using LDA embeddings, but not when computed using BERT embeddings. This indicates that the LDA embeddings are capturing specific "matches" between individual quiz questions and relatively well-defined time periods of lecture content, whereas the BERT embeddings appear to blur these specific correspondences. Taken together, these comparisons suggest that our LDA model trained specifically on the two lectures' contents is sensitive to subtle differences between the concepts probed by individual quiz questions, and can identify the relevant periods in the lectures where those concepts where presented (Fig. 4, Supp. Figs. 3, 4, Supp. Tab. 3). Meanwhile, BERT, whose embedding weights must be able to represent a far broader range of concepts, consequently blurs these subtle distinctions between concepts conveyed within the span of a single course lecture, thereby affording a "lower-resolution" depiction of its contents.

We also compared the within-lecture question matches identified by our LDA-based approach to analogous matches obtained via a simpler approach that did *not* entail using a text embedding model. Instead of projecting the text of the lectures and quiz questions into a common space and relating them by the similarities (i.e., correlations) between their coordinates (i.e., topic vectors), in this approach we related them by a measure of similarity computed directly from their texts. Specifically, we first parsed the two lectures' transcripts into overlapping sliding windows and performed the same preprocessing as we did for our LDA-based approach (see *Constructing text embeddings of multiple lectures and questions*). We then computed the Jaccard similarity between the text of each lecture-related quiz question and each sliding window from its corresponding lecture's transcript. Here, the Jaccard similarity between a question Q and a lecture window W is defined as the number of unique words that appear in the texts of both Q and W (i.e., $Q \cap W$) divided by the number of unique words that appear in either Q or W (i.e., $Q \cup W$).

As shown in Supplementary Figure 11, this simple word-matching approach does not appear to capture the same level of specificity as our LDA-based approach. Panels A and B display each question's distribution of topic vector correlations with all lecture timepoints (blue dots) alongside its distribution of Jaccard similarities to all sliding windows (orange dots). In these panels, both similarity measures have been normalized (independently, and separately for each question) to range between 0 and 1, such that a similarity of 1 reflects each question's "best matching" sample of lecture content, and a similarity of 0 reflects the least similar lecture content. In most cases, the distributions of topic vector correlations are dominated by extreme values (i.e., values close to 0 or 1) with comparatively few intermediate values. In other words, this approach yields strong and specific matches: each question's similarity is near its maximum value for a particular set of timepoints, and tends to be close to its minimum value for most other timepoints. The distributions of Jaccard similarities show the opposite pattern, consisting primarily of intermediate values with very few extreme values. In other words, this approach tends to weakly match each question to a large proportion of the lecture content.

These patterns are reflected in the similarity time series plots shown in panels C and D (analogous to main text Fig. 4). The questions' time series of topic vector correlations exhibit visually distinct "peaks" during specific sections of the lectures, outside of which their correlations tend to be relatively low. In other words, the set of timepoints whose topic vectors are maximally correlated with the topic vector for a given question tend to be not only specific, but also temporally contiguous. This indicates that topic vector correlation matches questions with sustained, cohesive periods of lecture content where the relevant underlying *concepts* are discussed, rather than with a sporadic assortment of timepoints where the question and lecture content share more superficial similarities. The time series of Jaccard similarities, by contrast, are less cleanly structured and do appear to reflect such superficial content matches in the more scattered, brief, and weak "peaks" they exhibit. As an example, panel E displays the text of the two quiz questions highlighted in green and purple in panels A through D, along with the text from the best-matching intervals from the lectures (identified based on topic vector correlation). Both questions include words that—given the subject matter of the two lectures—appear frequently throughout their transcripts

(e.g., “*force*”, “*atom*”, “*nucleus*”, “*cloud*”, “*dense*”, etc.). While the presence or absence of any one (or more) of these words will lead to differences in the degree of “match” based on Jaccard similarity (resulting in the “jagged” time series shown in panel D), LDA is able to capture some notion of the *context* in which different usages of the same words occur. As a result, the model is able to successfully assign similar topic vectors to quiz questions and lecture timepoints specifically when their shared combinations of words denote the same deeper meaning.

Supplementary references

[1] Blei, D. M., Ng, A. Y., and Jordan, M. I. (2003). Latent dirichlet allocation. *Journal of Machine Learning Research*, 3:993–1022.

[2] Devlin, J., Chang, M.-W., Lee, K., and Toutanova, K. (2018). BERT: pre-training of deep bidirectional transformers for language understanding. *arXiv*, 1810.04805.

[3] Touvron, H., Lavril, T., Izacard, G., Martinet, X., Lachaux, M.-A., Lacroix, T., Rozière, B., Goyal, N., Hambro, E., Azhar, F., Rodriguz, A., Joulin, A., Grave, E., and Lample, G. (2023). LLaMA: open and efficient foundation language models. *arXiv*, 2302.13971.

[4] Viswani, A., Shazeer, N., Parmar, N., Uszkoreit, J., Jones, L., Gomez, A. N., Kaiser, L., and Polosukhin, I. (2017). Attention is all you need. In *Advances in Neural Information Processing Systems*.
