# Supplementary material for: Text embedding models yield detailed conceptual knowledge maps derived from short multiple-choice quizzes
Source: Nat Commun. 2026 Mar 24;17:2055. doi: 10.1038/s41467-026-69746-w (PMC13013618; doi:10.1038/s41467-026-69746-w)
Supplement: Supplementary file 2 — Reporting Summary [file 41467_2026_69746_MOESM2_ESM.pdf]

nature portfolio

Corresponding author(s): Jeremy R. Manning

Last updated by author(s): Dec 1, 2025

Reporting Summary

Nature Portfolio wishes to improve the reproducibility of the work that we publish. This form provides structure for consistency and transparency in reporting. For further information on Nature Portfolio policies, see our Editorial Policies and the Editorial Policy Checklist.

Statistics

For all statistical analyses, confirm that the following items are present in the figure legend, table legend, main text, or Methods section.

n/a	Confirmed	
☐	☒	The exact sample size (n) for each experimental group/condition, given as a discrete number and unit of measurement
☐	☒	A statement on whether measurements were taken from distinct samples or whether the same sample was measured repeatedly
☐	☒	The statistical test(s) used AND whether they are one- or two-sided<br>*Only common tests should be described solely by name; describe more complex techniques in the Methods section.*
☐	☒	A description of all covariates tested
☐	☒	A description of any assumptions or corrections, such as tests of normality and adjustment for multiple comparisons
☐	☒	A full description of the statistical parameters including central tendency (e.g. means) or other basic estimates (e.g. regression coefficient) AND variation (e.g. standard deviation) or associated estimates of uncertainty (e.g. confidence intervals)
☐	☒	For null hypothesis testing, the test statistic (e.g. F, t, r) with confidence intervals, effect sizes, degrees of freedom and P value noted<br>*Give P values as exact values whenever suitable.*
☒	☐	For Bayesian analysis, information on the choice of priors and Markov chain Monte Carlo settings
☐	☒	For hierarchical and complex designs, identification of the appropriate level for tests and full reporting of outcomes
☐	☒	Estimates of effect sizes (e.g. Cohen's d, Pearson's r), indicating how they were calculated

Our web collection on statistics for biologists contains articles on many of the points above.

Software and code

Policy information about availability of computer code

Data collection

All code used to collect the data in this study, a Dockerfile for reproducing the original experiment environment, and instructions for building the environment and running the experiment are available at https://github.com/ContextLab/efficient-learning-khan. The experiment uses psiTurk v3.3.1 (https://github.com/NYUCCL/psiTurk) and jsPsych v6.0.0 (https://github.com/jspsych/jsPsych).

Data analysis

All data analysis code used in this study, a Dockerfile for reproducing the analysis environment, and instructions for building the environment and running the analyses are available at https://github.com/ContextLab/efficient-learning-khan. All data analysis code was written using Python v3.9 (https://www.python.org) and open-source Python libraries. Names and exact versions of all libraries used in the analyses may be found at https://github.com/ContextLab/efficient-learning-khan/blob/master/docker/analysis-environment.yml and are additionally listed below:
_openmp_mutex v4.5
_r-mutex v1.0.1
_sysroot_linux-aarch64_curr_repodata_hack v4
argon2-cffi v21.3.0
argon2-cffi-bindings v21.2.0
asttokens v2.0.5
attrs v21.4.0
backcall v0.2.0
backports v1.0
backports.functools_lru_cache v1.6.4
beautifulsoup4 v4.11.1
binutils_impl_linux-aarch64 v2.36.1

black v22.3.0
bleach v5.0.0
brotli v1.0.9
brotli-bin v1.0.9
brotlipy v0.7.0
bwidget v1.9.14
bzip2 v1.0.8
c-ares v1.18.1
ca-certificates v2024.2.2
cairo v1.16.0
certifi v2024.2.2
cffi v1.15.0
charset-normalizer v2.0.9
click v8.1.2
colorama v0.4.4
conda v4.11.0
conda-package-handling v1.7.3
cryptography v42.0.2
curl v8.1.2
cycler v0.11.0
dataclasses v0.8
debugpy v1.6.0
decorator v5.1.1
defusedxml v0.7.1
entrypoints v0.4
executing v0.8.3
expat v2.5.0
flit-core v3.7.1
fmt v9.1.0
font-ttf-dejavu-sans-mono v2.37
font-ttf-inconsolata v3.000
font-ttf-source-code-pro v2.038
font-ttf-ubuntu v0.83
fontconfig v2.14.2
fonts-conda-ecosystem v1
fonts-conda-forge v1
fonttools v4.32.0
freetype v2.12.1
fribidi v1.0.10
gcc_impl_linux-aarch64 v12.1.0
gettext v0.21.1
gfortran_impl_linux-aarch64 v12.1.0
giflib v5.2.1
graphite2 v1.3.13
gsl v2.7
gxx_impl_linux-aarch64 v12.1.0
harfbuzz v6.0.0
icu v70.1
idna v3.1
importlib-metadata v4.11.3
importlib_resources v5.7.1
ipykernel v6.13.0
ipython v8.0.1
ipython_genutils v0.2.0
ipywidgets v7.7.0
jbig v2.1
jedi v0.18.1
jinja2 v3.1.1
joblib v1.1.0
jpeg v9e
jsonschema v4.4.0
jupyter_client v7.2.2
jupyter_contrib_core v0.3.3
jupyter_contrib_nbextensions v0.5.1
jupyter_core v4.9.2
jupyter_highlight_selected_word v0.2.0
jupyter_latex_envs v1.4.6
jupyter_nbextensions_configurator v0.4.1
jupyterlab_pygments v0.2.2
jupyterlab_widgets v1.1.0
kernel-headers_linux-aarch64 v4.18.0
keyutils v1.6.1
kiwisolver v1.4.2
krb5 v1.20.1
lcms2 v2.15
ld_impl_linux-aarch64 v2.36.1

lerc v4.0.0
libarchive v3.6.2
libblas v3.9.0
libbrotlicommon v1.0.9
libbrotlidec v1.0.9
libbrotlienc v1.0.9
libcblas v3.9.0
libcurl v8.1.2
libdeflate v1.17
libedit v3.1.20191231
libev v4.33
libexpat v2.5.0
libffi v3.4.2
libgcc-devel_linux-aarch64 v12.1.0
libgcc-ng v13.2.0
libgfortran-ng v13.2.0
libgfortran5 v13.2.0
libglib v2.78.1
libgomp v13.2.0
libiconv v1.17
liblapack v3.9.0
libllvm10 v10.0.1
libmamba v1.1.0
libmambapy v1.1.0
libnghttp2 v1.52.0
libnsl v2.0.0
libopenblas v0.3.20
libpng v1.6.42
libsanitizer v12.1.0
libsodium v1.0.18
libsolv v0.7.28
libsqlite v3.44.2
libssh2 v1.11.0
libstdcxx-devel_linux-aarch64 v12.1.0
libstdcxx-ng v13.2.0
libtiff v4.5.0
libuuid v2.38.1
libwebp v1.2.4
libwebp-base v1.2.4
libxcb v1.13
libxml2 v2.10.3
libxslt v1.1.37
libzlib v1.2.13
llvmlite v0.36.0
lxml v4.8.0
lz4-c v1.9.3
lzo v2.10
make v4.3
mamba v1.1.0
markupsafe v2.1.1
matplotlib v3.5.1
matplotlib-base v3.5.1
matplotlib-inline v0.1.3
mistune v0.8.4
munkres v1.1.4
mypy_extensions v0.4.3
nbclient v0.6.0
nbconvert v6.5.0
nbconvert-core v6.5.0
nbconvert-pandoc v6.5.0
nbformat v5.3.0
ncurses v6.4
nest-asyncio v1.5.5
nlopt v2.7.1
nltk v3.6.7
notebook v6.4.7
numba v0.53.1
numpy v1.20.3
openjpeg v2.5.0
openssl v3.2.1
packaging v21.3
pandas v1.3.5
pandoc v2.18
pandocfilters v1.5.0
pango v1.50.14
parso v0.8.3

pathspec v0.9.0
patsy v0.5.2
pcre2 v10.40
pexpect v4.8.0
pickleshare v0.7.5
pillow v9.4.0
pip v21.3.1
pixman v0.43.2
platformdirs v2.5.1
prometheus_client v0.14.1
prompt-toolkit v3.0.29
psutil v5.9.0
pthread-stubs v0.4
ptyprocess v0.7.0
pure_eval v0.2.2
pybind11-abi v4
pycosat v0.6.3
pycparser v2.21
pygments v2.11.2
ejolly::pymer4 v0.8.1
pynndescent v0.5.6
pyopenssl v24.0.0
pyparsing v3.0.8
pyrsistent v0.18.1
pysocks v1.7.1
python v3.9.18
python-dateutil v2.8.2
python-fastjsonschema v2.15.3
python_abi v3.9
pytz v2022.1
pyyaml v6.0.1
pyzmq v22.3.0
r-backports v1.4.1
r-base v4.2.3
r-boot v1.3_28.1
r-broom v1.0.5
r-cli v3.6.2
r-colorspace v2.1_0
r-crayon v1.5.2
r-datawizard v0.9.1
r-dplyr v1.1.4
r-ellipsis v0.3.2
r-emmeans v1.10.0
r-estimability v1.4.1
r-evaluate v0.23
r-fansi v1.0.6
r-farver v2.1.1
r-generics v0.1.3
r-ggeffects v1.2.1
r-ggplot2 v3.4.4
r-glue v1.7.0
r-gtable v0.3.4
r-highr v0.10
r-insight v0.19.8
r-isoband v0.2.7
r-knitr v1.45
r-labeling v0.4.3
r-lattice v0.20_45
r-lifecycle v1.0.4
r-lme4 v1.1_32
r-lmertest v3.1_3
r-magrittr v2.0.3
r-mass v7.3_58.2
r-matrix v1.5_3
r-mgcv v1.9_1
r-minqa v1.2.5
r-munsell v0.5.0
r-mvtnorm v1.2_4
r-nlme v3.1_162
r-nloptr v2.0.3
r-numderiv v2016.8_1.1
r-pbkrtest v0.5.2
r-pillar v1.9.0
r-pkgconfig v2.0.3
r-purrr v1.0.2
r-r6 v2.5.1

r-rcolorbrewer v1.1_3
r-rcpp v1.0.10
r-rcppeigen v0.3.3.9.4
r-rlang v1.1.3
r-scales v1.3.0
r-sjlabelled v1.2.0
r-statmod v1.5.0
r-stringi v1.7.12
r-stringr v1.5.1
r-tibble v3.2.1
r-tidyr v1.3.1
r-tidyselect v1.2.0
r-utf8 v1.2.4
r-vctrs v0.6.5
r-viridislite v0.4.2
r-withr v3.0.0
r-xfun v0.42
r-xtable v1.8_4
r-yaml v2.3.8
readline v8.2
regex v2022.3.15
reproc v14.2.3
reproc-cpp v14.2.3
requests v2.26.0
rpy2 v3.5.11
ruamel_yaml v0.15.80
scikit-learn v1.0.2
scipy v1.8.0
seaborn v0.11.2
seaborn-base v0.11.2
sed v4.8
send2trash v1.8.0
setuptools v59.4.0
simplegeneric v0.8.1
six v1.16.0
soupsieve v2.3.1
sqlite v3.44.2
stack_data v0.2.0
statsmodels v0.13.2
sysroot_linux-aarch64 v2.17
tbb v2020.2
terminado v0.13.3
threadpoolctl v3.1.0
tinycss2 v1.1.1
tk v8.6.13
tktable v2.10
tomli v2.0.1
tornado v6.1
tqdm v4.62.3
traitlets v5.1.1
typed-ast v1.5.3
typing_extensions v4.2.0
tzdata v2021e
tzlocal v5.2
umap-learn v0.5.2
unicodedata2 v14.0.0
urllib3 v1.26.7
wcwidth v0.2.5
webencodings v0.5.1
wheel v0.37.0
widgetsnbextension v3.6.0
xorg-kbproto v1.0.7
xorg-libice v1.0.10
xorg-libsm v1.2.3
xorg-libx11 v1.8.4
xorg-libxau v1.0.9
xorg-libxdmcp v1.1.3
xorg-libxext v1.3.4
xorg-libxrender v0.9.10
xorg-libxt v1.3.0
xorg-renderproto v0.11.1
xorg-xextproto v7.3.0
xorg-xproto v7.0.31
xz v5.2.6
yaml v0.2.5
yaml-cpp v0.7.0

zeromq v4.3.4
zipp v3.8.0
zlib v1.2.13
zstd v1.5.5

For manuscripts utilizing custom algorithms or software that are central to the research but not yet described in published literature, software must be made available to editors and reviewers. We strongly encourage code deposition in a community repository (e.g. GitHub). See the Nature Portfolio guidelines for submitting code & software for further information.

Data

Policy information about availability of data

All manuscripts must include a data availability statement. This statement should provide the following information, where applicable:

- Accession codes, unique identifiers, or web links for publicly available datasets
- A description of any restrictions on data availability
- For clinical datasets or third party data, please ensure that the statement adheres to our policy

All data analyzed in this manuscript may be found at https://github.com/ContextLab/efficient-learning-khan.

Research involving human participants, their data, or biological material

Policy information about studies with human participants or human data. See also policy information about sex, gender (identity/presentation), and sexual orientation and race, ethnicity and racism.

Reporting on sex and gender	A total of 15 participants self-reported their gender as male; 35 participants self-reported their gender as female. Neither sex nor gender was considered in the study design. Beyond demographics reporting, neither sex nor gender was considered in data analyses as we had no a priori hypotheses about sex or gender differences in this study.
Reporting on race, ethnicity, or other socially relevant groupings	A total of 47 participants self-reported their ethnicity as "Not Hispanic or Latino" and three reported their ethnicity as "Hispanic or Latino." Participants self-reported their races as "White" (32 participants), "Asian" (14 participants), "Black or African American" (5 participants), "American Indian or Alaska Native" (1 participant), and "Native Hawaiian or Other Pacific Islander" (1 participant). Neither race nor ethnicity was considered in the study design. Beyond demographics reporting, neither race nor ethnicity was considered in data analyses as we had no a priori hypotheses about racial or ethnic differences in this study.
Population characteristics	See below
Recruitment	Participants were recruited from introductory-level courses in the Department of Psychological and Brain Sciences at Dartmouth College via the SONA Systems platform. Participants received optional course credit for enrolling and provided informed consent prior to the start of the experiment.
Ethics oversight	Our experimental protocol was approved by the Committee for the Protection of Human Subjects at Dartmouth College.

Note that full information on the approval of the study protocol must also be provided in the manuscript.

Field-specific reporting

Please select the one below that is the best fit for your research. If you are not sure, read the appropriate sections before making your selection.

☐ Life sciences ☒ Behavioural & social sciences ☐ Ecological, evolutionary & environmental sciences

For a reference copy of the document with all sections, see nature.com/documents/nr-reporting-summary-flat.pdf

Behavioural & social sciences study design

All studies must disclose on these points even when the disclosure is negative.

Study description	The study was quantitative and used a repeated-measures design. Data collected comprised participants' performance on simple multiple-choice quizzes before, between, and after viewing two brief course lectures.
Research sample	Participants comprised 50 undergraduate students at Dartmouth College, ranging in age from 18 to 22 years (mean: 19.52 years; standard deviation: 1.09 years). A total of 49 participants self-reported their native language as "English"; 1 participant self-reported having another native language. A total of 15 participants self-reported their gender as male; 35 participants self-reported their gender as female. A total of 3 participants self-reported their ethnicity as "Hispanic or Latino"; 47 self-reported their ethnicity as "Not Hispanic or Latino." Participants self-reported their races as "White" (32 participants), "Asian" (14 participants), "Black or African American" (5 participants), "American Indian or Alaska Native" (1 participant), and "Native Hawaiian or Other Pacific Islander" (1 participant). (Note that some participants selected multiple racial categories.)
Sampling strategy	The study used a convenience sample. Participants were recruited from introductory-level courses in the Department of Psychological and Brain Sciences at Dartmouth College via the SONA Systems platform. Due to the novelty of the analysis framework,

	no statistical tests were used to predetermine sample size; however, the sample size was chosen to be comparable to that of similar classroom learning-based experimental paradigms.
Data collection	The experiment was conducted in a light- and sound-attenuated testing room. Lecture videos and multiple-choice quizzes were presented on a 27-inch 2015 iMac desktop computer (resolution: 5120 x 2880) with lecture audio played through the computer's built-in speakers. Participants used an Apple Magic Trackpad to select answers to multiple-choice questions and progress between lecture videos and quizzes. The researcher consented and debriefed participants but was not present during the experimental session and was blind to individual participants' quiz performances. The experiment was built using the psiTurk platform and the jsPsych library.
Timing	Data were collected between 26 April 2019 and 25 October 2019
Data exclusions	No data were excluded from the analyses.
Non-participation	No participants dropped out or declined participation.
Randomization	Participants were not allocated into experimental groups. The 13 questions appearing on each of the three quizzes were randomly selected (without replacement) from the overall pool of 39 questions, for each participant, separately. This randomization was constrained such that each quiz contain exactly 5 questions about Lecture 1, 5 questions about Lecture 2, and 3 questions about general physics concepts. The order of the 13 questions appearing on each quiz and the order of answer options for each multiple-choice question were also randomized.

Reporting for specific materials, systems and methods

We require information from authors about some types of materials, experimental systems and methods used in many studies. Here, indicate whether each material, system or method listed is relevant to your study. If you are not sure if a list item applies to your research, read the appropriate section before selecting a response.

Materials & experimental systems

n/a	Involved in the study
☒	☐ Antibodies
☒	☐ Eukaryotic cell lines
☒	☐ Palaeontology and archaeology
☒	☐ Animals and other organisms
☒	☐ Clinical data
☒	☐ Dual use research of concern
☒	☐ Plants

Methods

n/a	Involved in the study
☒	☐ ChIP-seq
☒	☐ Flow cytometry
☒	☐ MRI-based neuroimaging

Plants

Seed stocks	*Report on the source of all seed stocks or other plant material used. If applicable, state the seed stock centre and catalogue number. If plant specimens were collected from the field, describe the collection location, date and sampling procedures.*
Novel plant genotypes	*Describe the methods by which all novel plant genotypes were produced. This includes those generated by transgenic approaches, gene editing, chemical/radiation-based mutagenesis and hybridization. For transgenic lines, describe the transformation method, the number of independent lines analyzed and the generation upon which experiments were performed. For gene-edited lines, describe the editor used, the endogenous sequence targeted for editing, the targeting guide RNA sequence (if applicable) and how the editor was applied.*
Authentication	*Describe any authentication procedures for each seed stock used or novel genotype generated. Describe any experiments used to assess the effect of a mutation and, where applicable, how potential secondary effects (e.g. second site T-DNA insertions, mosiacism, off-target gene editing) were examined.*
